# Unexpected Orange Photoluminescence from Tetrahedral Manganese(II) Halide Complexes with Bidentate Phosphanimines

**DOI:** 10.3390/molecules31010161

**Published:** 2026-01-01

**Authors:** Domenico Piccolo, Jesús Castro, Valentina Beghetto, Daniele Rosa-Gastaldo, Marco Bortoluzzi

**Affiliations:** 1 Dipartimento di Scienze Molecolari e Nanosistemi, Università Ca’ Foscari Venezia, 30172 Mestre, Italy; domenico.piccolo@unive.it (D.P.); beghetto@unive.it (V.B.); 2 Dipartimento di Scienze Chimiche, Università di Padova, Via Marzolo 1, 35131 Padova, Italy; daniele.rosagastaldo@unipd.it; 3 Departamento de Química Inorgánica, Facultade de Química, Universidade de Vigo, Edificio de Ciencias Experimentais, 36310 Vigo, Spain; jesusc@uvigo.gal; 4 CIRCC (Consorzio Universitario Reattività Chimica e Catalisi), Via Celso Ulpiani 27, 70126 Bari, Italy

**Keywords:** manganese, tetrahedral complexes, halide complexes, phosphanimines, photoluminescence

## Abstract

Manganese(II) halide complexes with the general formula [MnX_2_{(PhN=PPh_2_)CH_2_}], where X is bromine or iodine and (PhN=PPh_2_)CH_2_ is the bis-phosphanimine ligand 1,1′-methylenebis-(*N*,1,1-triphenylphosphanimine), were prepared and isolated. The structure of the two compounds was determined by single-crystal X-ray diffraction, revealing an approximately tetrahedral geometry at the metal centre. Unlike structurally comparable compounds containing phosphine oxides or related [O=P]-donors in the coordination sphere, which commonly show green emissions, solid samples of [MnBr_2_{(PhN=PPh_2_)CH_2_}] and [MnI_2_{(PhN=PPh_2_)CH_2_}] exhibited orange emissions upon irradiation with UV light. The emission spectra resulted excitation-independent. Superimposable steady-state luminescence spectra were collected for both compounds as powders and crystals suitable for X-ray diffraction. The excitation spectra and the ligand→metal antenna effect were affected by the coordinated halide, and only [MnBr_2_{(PhN=PPh_2_)CH_2_}] showed bright luminescence under near-UV irradiation. Either ligand- or metal-centred transitions can account for the observed luminescence, and the luminescence decay curves were consistent with a multiplicity change from the excited to the ground state, with excited-state lifetimes in the range of hundreds of microseconds. Attempts to rationalize the unexpected luminescence were carried out based on DFT calculations.

## 1. Introduction

Phosphanimines are compounds of pentavalent phosphorus characterized by versatile coordination behaviour, as revealed since the 1960s by the preparation of several d-block metal complexes from Group 4 to Group 12. Selected examples of structurally characterized coordination compounds with monodentate phosphanimines are the halide complexes [CrCl_2_(Me_3_SiN=PMe_3_)_2_] [1], [CoCl_2_(RN=PMe_3_)_2_] (R = H, SiMe_3_) [2], [RhCl(COD)(CH_3_C_6_H_4_N=PEt_3_)] (COD = 1,5-cyclooctadiene; CH_3_C_6_H_4_ = *p*-tolyl) [3], [PdCl_2_(Me_3_SiN=PEt_3_)_2_] [1], [CuCl(PhN=PPh_3_)] [4], [Cu_2_Cl_2_(μ-Cl)_2_(Me_3_SiN=PR_3_)_2_] (R = Me, Ph) [1,5], [ZnCl_2_(Me_3_SiN=PMe_3_)_2_] [2], [CdI_2_(HN=PPh_3_)_2_] [6], and [Hg_2_Cl_2_(μ-Cl)_2_(PhN=PPh_3_)_2_] [7]. Linear copper(I) derivatives with the general formula [Cu(phosphanimine)_2_]^+^ are examples of homoleptic phosphanimine coordination compounds, and they have been found to be active catalysts for azide–alkyne coupling [8].

The presence of suitable substituents in the phosphanimine structure can afford chelating ligands. Polydentate phosphanimin-phosphines have found application in the synthesis of homogeneous catalysts active in reactions such as hydrogenation, hydrogen transfer, cross-coupling, oligomerization, and cyclization [9]. An example of structurally characterized homogeneous catalyst is [NiBr_2_(^i^PrN=PPh_2_–C_6_H_4_–PPh_2_)], where the N^P donor is 2-[propan-2-ylimino(diphenyl)-λ^5^-phosphanyl]phenyl(diphenyl)phosphine, able to catalyze the dimerization of ethene [10].

Several poly(phosphanimines) with donor fragments connected through either the nitrogen or the phosphorus atoms have been synthesized and applied in the preparation of d-block coordination compounds. An example of a complex with a bidentate phosphanimine characterized by X-ray diffraction is the rhodium(I) derivative [Rh{(CH_3_C_6_H_4_N=PPh_2_)_2_CH_2_}(COD)]^+^ [11]. Coordination compounds featuring tridentate ligands with at least one phosphanimine donor moiety are summarized in a recent review [12]. It is worth noting that bidentate phosphanimines in which the {RN=P} moieties are connected through a P-bonded methylene bridge are precursors for the preparation of related bis(phosphanimine)methanide metal complexes. The anionic ligands are stabilized by π-delocalization over the N_2_P_2_C framework and by σ-bonding between the CHP_2_ carbon atom and the metal centre. For instance, the reaction of NiBr_2_(DME) (DME = 1,2-dimethoxyethane) with (Ph^iPr2^N=PPh_2_)_2_CH_2_ afforded the paramagnetic complex [NiBr_2_{(Ph^iPr2^N=PPh_2_)_2_CH_2_}], while the reaction of the metal precursor with the conjugate base of the ligand led to the formation of the diamagnetic square-planar derivative [NiBr{(Ph^iPr2^N=PPh_2_)_2_CH}] [13,14].

The {RN=P} group of phosphanimines is best described as a resonance hybrid between the two forms {NR=P} and {RN^−^–P^+^}, with the second form gaining importance after the formation of the M–N bond [15]. The electronic features are in part comparable to those of the {O=P} fragment in phosphine oxides. Ligands containing this donor moiety are used for the preparation of luminescent manganese(II) complexes of interest in the field of advanced optics. For instance, the coordination of monodentate phosphine oxides to manganese(II) halides afforded tetrahedral coordination compounds characterized by bright green photo- and triboluminescence [16,17,18,19,20]. The wavelength of the Mn(II) emission is primarily determined by the coordination geometry [21,22], and the use of chelating bidentate phosphine oxides can lead to manganese(II) complexes with variable luminescence. Highly luminescent green-emitting tetrahedral species were reported [23,24,25,26,27,28], while metal-centred emissions in the orange–red range were observed for five-coordinated [29,30,31,32] and octahedral complexes [33,34,35,36]. The scenario broadens when considering that structural changes can be induced by varying the experimental conditions, and that bidentate phosphine oxides can behave as bridging ligands between two manganese(II) centres, with the formation of polynuclear coordination compounds [37,38,39,40].

The chemistry of manganese(II) with neutral phosphanimine ligands has been comparatively less studied. The complexes [Mn_2_Cl_2_(μ-Cl)_2_(Me_3_SiN=PEt_3_)_2_] and [MnI_2_(Me_3_SiN=PEt_3_)_2_] were prepared through the reaction of Me_3_SiN=PEt_3_ with the appropriate manganese(II) halide and structurally characterized. Thermal treatment of [MnI_2_(Me_3_SiN=PEt_3_)_2_] afforded the chelate complex [MnI_2_{(Et_3_P=N)_2_SiMe_2_}] [41]. Other manganese(II) complexes are bis(phosphanimine)methanide derivatives with the general formula [Mn{N(SiMe_3_)_2_}{(ArN=PPh_2_)CH}] (Ar = substituted aryl), formed by reacting the corresponding neutral bis(phosphanimine) with the bis(amido) precursor [Mn{N(SiMe_3_)_2_}_2_] [42,43]. These compounds were not investigated from a photophysical point of view.

**Figure 1:**
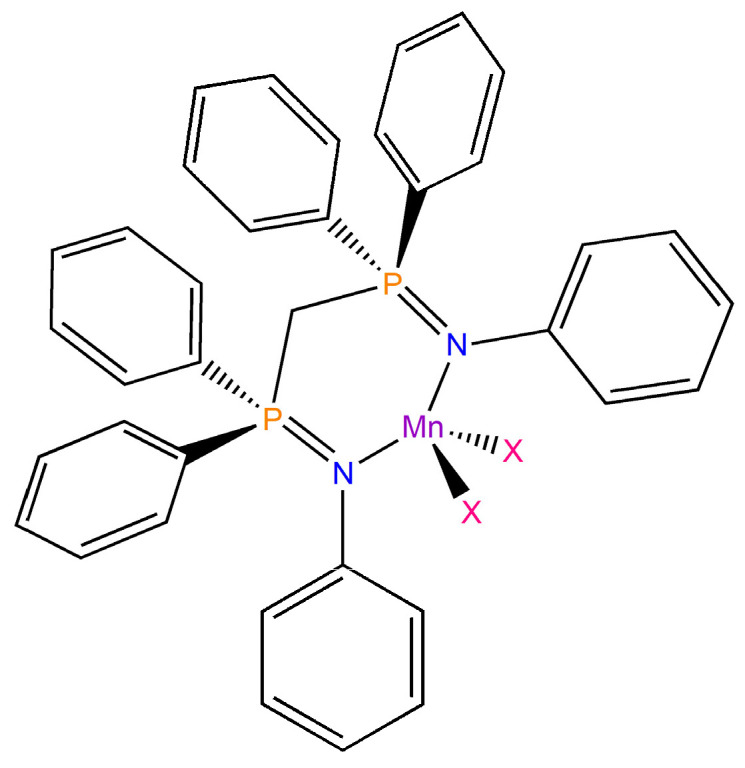
[MnX_2_{(PhN=PPh_2_)CH_2_}] (X = Br, I).

Manganese(II) phosphaniminine complexes with the general formula [MnX_2_(NPh=PPh_3_)_2_] and organic–inorganic hybrids of the type [NHPh=PPh_3_]_2_[MnX_4_] (X = Cl, Br, I) were recently synthesized, and steady-state and time-resolved photoluminescence measurements were carried out [44]. In the present work, the study was extended to complexes with the general formula [MnX_2_{(PhN=PPh_2_)CH_2_}], where X is bromine or iodine and (PhN=PPh_2_)CH_2_ is the bis(phosphanimine) ligand 1,1′-methylenebis-(*N*,1,1-triphenylphosphanimine (Figure 1). The structures of the two compounds were determined by single-crystal X-ray diffraction, revealing an approximately tetrahedral geometry at the metal centre. Unlike structurally comparable compounds containing phosphine oxides in the coordination sphere, solid samples of [MnBr_2_{(PhN=PPh_2_)CH_2_}] and [MnI_2_{(PhN=PPh_2_)CH_2_}] exhibited bright orange emissions upon irradiation with near-UV light. The luminescence of the new complexes is discussed in the next sections.

## 2. Results and Discussion

### 2.1. Synthesis, Characterization, and Single-Crystal X-Ray Diffraction of the Complexes

The reaction of anhydrous MnX_2_ halides with a stoichiometric amount of (PhN=PPh_2_)CH_2_ in acetonitrile at room temperature afforded the complexes [MnX_2_{(PhN=PPh_2_)CH_2_}] (X = Br, I) with good yields. Elemental analysis data agree with the proposed formulations. The molar magnetic susceptibility values calculated using the [MnX_2_{(PhN=PPh_2_)CH_2_}] molecular weights were in line with high-spin d^5^ first-row transition metal centres (magnetic moments around 5.9 BM at 293 K).

**Figure 2:**
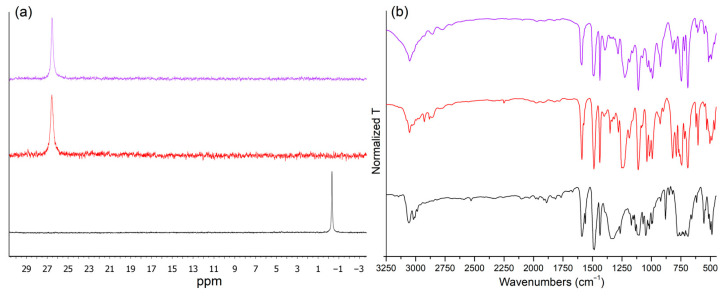
^31^P{^1^H} NMR (CDCl_3_, 300 K) (**a**) and FT-IR (KBr) (**b**) spectra of (PhN=PPh_2_)CH_2_ (black lines), [MnBr_2_{(PhN=PPh_2_)CH_2_}] (red lines), and [MnI_2_{(PhN=PPh_2_)CH_2_}] (violet lines).

The ^31^P{^1^H} NMR spectra of both compounds in CDCl_3_ at 300 K were composed of a single resonance at 26.6 ppm, with full width at half maximum (FWHM) values between 35 and 50 Hz. The ^31^P resonance is noticeably shifted to a higher frequency compared to the free ligand (δ = −0.3 ppm under the same experimental conditions), probably because the interaction with MnX_2_ increases the relative weight of the (PhN^−^–^+^PPh_2_)_2_CH_2_ charge-separation ylide form. Moreover, the ^31^P resonance in free (PhN=PPh_2_)CH_2_ is sharper (FWHM = 15 Hz) thanks to the lack of paramagnetic centres (Figure 2). The ^1^H NMR spectra of the complexes were less diagnostic because of the broadness of the signals caused by paramagnetic relaxation (Figure S1), and thus it was not possible to assign the resonance corresponding to the methylene bridge (triplet, δ = 3.72 ppm, *J*_PH_ = 13.4 Hz in the free ligand). Coordination effects on the phosphorus–nitrogen bonds are also evident comparing the FT-IR spectra of the free ligand and of the corresponding [MnX_2_{(PhN=PPh_2_)CH_2_}] complexes (Figure 2). In particular, the wide band centred at 1330 cm^−1^ in the FT-IR spectrum of (PhN=PPh_2_)CH_2_, assigned to ν_PN_ stretchings, is noticeably shifted to lower wavenumbers in the manganese(II) derivatives, falling in the 1250–1220 cm^−1^ range. Similar results were obtained by comparing the FT-IR spectra of the monodentate phosphanimine NPh=PPh_3_ and of the corresponding [MnX_2_(NPh=PPh_3_)_2_] (X = Cl, Br, I) complexes [44]. The AIM analysis [45] of the (3,−1) N=P bond critical points (BCPs) in the DFT-optimized structures of (PhN=PPh_2_)CH_2_ and of its Mn(II) complexes confirmed that the strength of the N=P bonds lowers upon coordination, with average values of potential energy density at BCP equal to −0.578 a.u. in the free ligand and −0.530 a.u. in [MnX_2_{(PhN=PPh_2_)CH_2_}].

The melting points of the two [MnX_2_{(PhN=PPh_2_)CH_2_}] complexes are similar, around 130 °C. TGA measurements under N_2_ atmosphere revealed that the two compounds start decomposing with mass loss at temperatures above 230 °C. The temperature threshold is strictly comparable with that obtained from TGA measurements on the free ligand (Figure S2).

**Figure 3:**
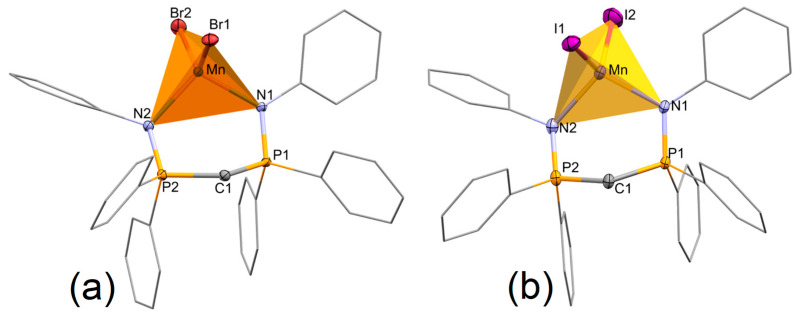
Structures of [MnBr_2_{(PhN=PPh_2_)CH_2_}] (**a**) and [MnI_2_{(PhN=PPh_2_)CH_2_}] (**b**). Selected bond lengths [Å] and angles [°]: **X = Br**, Mn-N(1) 2.117(2), Mn-N(2) 2.126(2), Mn-Br(1) 2.4943(5), Mn-Br(2) 2.4659(5), P(1)-N(1) 1.605(2), P(2)-N(2) 1.598(2), N(1)-Mn-N(2) 104.65(9), N(1)-Mn-Br(2) 106.64(6), N(2)-Mn-Br(2) 110.22(6), N(1)-Mn-Br(1) 103.61(7), N(2)-Mn-Br(1) 102.88(6), Br(1)-Mn-Br(2) 126.82(2); **X = I**, Mn-N(1) 2.109(4), Mn-N(2) 2.114(4), Mn-I(1) 2.6583(8), Mn-I(2) 2.6069(9), P(1)-N(1) 1.603(4), P(2)-N(2) 1.597(4), N(1)-Mn-N(2) 105.26(15), N(1)-Mn-I(2) 106.95(12), N(2)-Mn-I(2) 110.17(12), N(1)-Mn-I(1) 104.97(12), N(2)-Mn-I(1) 103.55(12), I(1)-Mn-I(2) 124.43(3).

The formation of mononuclear complexes with distorted tetrahedral geometry was confirmed by X-ray diffraction on single crystals grown by slow diffusion of diethyl ether in dichloromethane solutions. Figure 3 shows the structures of the two compounds (see Figure S3 for their superimposition). Selected descriptors for the four-coordinate geometry are collected in Table S1. The differences between the two complexes are scarce, mainly related to the different lengths of the Mn–X bonds. In both compounds, there is a dichloromethane crystallization molecule that was removed from the plots shown in Figure 3. The manganese ion is tetracoordinated to two halide atoms and two nitrogen atoms of the bidentate (PhN=PPh_2_)CH_2_ ligand. Selected distances and angles are reported in the caption of Figure 3. The Mn–X bond lengths are in agreement with the change in the halogen, following the expected trend that Mn–Br bonds are shorter than Mn–I bonds [23,46]. The Mn–N distances become shorter on increasing the Mn–X bond length [X = Br, 2.117(2) and 2.126(2) Å, X = I, 2.109(4) and 2.114(4)]. The values are in line with those observed for other manganese(II) phosphanimine complexes [41] and for the bis(phosphanimine)methanide complex [Mn{N(SiMe_3_)_2_}{(Ph^Me3^N=PPh_2_)CH}] (Ph^Me3^ = mesityl) [42]. The Mn–N bond lengths are also comparable to those found in the phosphoraneiminato cubane complex [MnBr(NPEt_3_)]_4_ [47].

The most obtuse angle is the X-Mn-X angle, as occurs in other [MnX_2_(L^L)] complexes where the bite angle is not constrained by the structure of the bridge between the donor moieties [23,24,25,26,27,28], and it is larger for X = Br. The N–Mn–N angles, 104.65(9) and 105.26(15)° for X = Br and X = I, respectively, seem to be scarcely affected by the bidentate character of the ligand and adopt values relatively close to the O–Mn–O angles observed for related halide complexes with monodentate [O=P]-donors [46,48,49]. The N–Mn–N is meaningfully larger [123.1(7)°] in the phosphanimine complex [MnI_2_(Me_3_SiNPEt_3_)_2_] [41], probably because of the high steric hindrance of the trimethylsilyl substituents.

The six-membered metallacycle adopts a non-planar conformation resulting in a somewhat distorted screw-boat geometry. The classical parameters to define the puckering [50] are set out in the caption of Figure S4, and they are in accordance with a definition of θ around 90.0° and φ around k X 60 + 30. The intra-ring torsion angles are shown in Figure S4 to allow for conformational analysis of the rings. A closely related metallacycle was found in the tricoordinated bis(phosphanimine)methanide complex [Mn{N(SiMe_3_)_2_}{(Ph^Me3^N=PPh_2_)CH}] [42], which shows slightly longer P–N bond distances (average 1.613 Å) compared to the complexes described here (average 1.601 Å). In the tricoordinated complex, the angles defined by the P(1)–C(1)–P(2) and N(1)–Mn–N(2) least-square planes and the P(1)–N(1)–P(2)–N(2) plane are, respectively, 61.25 and 54.36°, whilst in the tetracoordinated [MnX_2_{(PhN=PPh_2_)CH_2_}] compounds, the angles are 55.90(12) and 20.25(16)° for X = Br and 55.26(20) and 18.1(3)° for X = I. The values reveal that the manganese(II) centre is much closer to the P_2_N_2_ plane than in the tricoordinated complex. It is, however, worth noting that the P_2_N_2_ best square plane is far from the planarity, as expected for a screw-boat geometry, with root mean square deviations of 0.1029 and 0.1132 Å and P(1)–N(1)–N(2)–P(2) torsion angles of 14.4(2) and 15.9(2)° for X = Br and X = I, respectively.

[MnBr_2_{(PhN=PPh_2_)CH_2_}] and [MnI_2_{(PhN=PPh_2_)CH_2_}] crystals are isomorphs, and both contain a dichloromethane molecule in the crystal structure. The intermolecular interactions have the same nature in the two compounds and are constituted by C–H---X hydrogen bonds between two [MnX_2_{(PhN=PPh_2_)CH_2_}] complexes and between one [MnX_2_{(PhN=PPh_2_)CH_2_}] molecule and CH_2_Cl_2_, together with the interaction of a chlorine atom with a P-bonded phenyl ring. Figure S5 shows these interactions for [MnBr_2_{(PhN=PPh_2_)CH_2_}]. The parameters for the intermolecular interactions are set out in Table S2.

### 2.2. Photoluminescence of the Complexes

**Figure 4:**
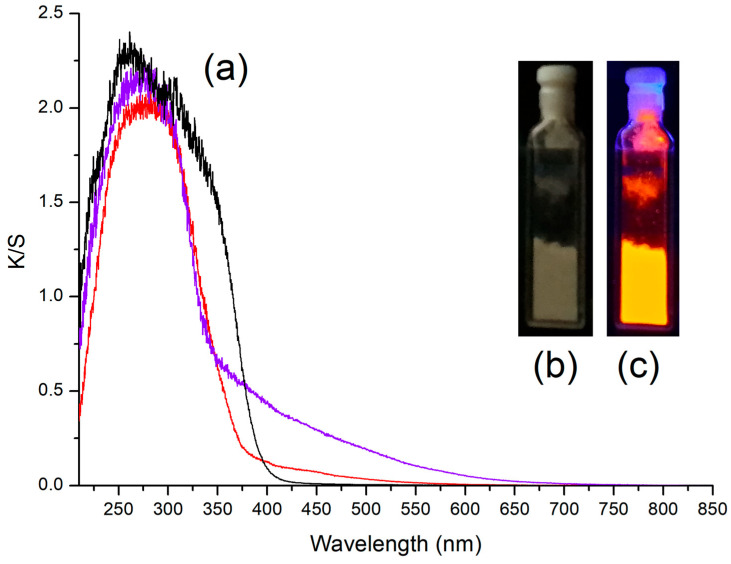
(**a**) Kubelka–Munk transformation of the reflectance spectra of (PhN=PPh_2_)CH_2_ (black line), [MnBr_2_{(PhN=PPh_2_)CH_2_}] (red line), and [MnI_2_{(PhN=PPh_2_)CH_2_}] (violet line) (**a**). Pictures of solid [MnBr_2_{(PhN=PPh_2_)CH_2_}] illuminated with indoor light (**b**) and UV light (**c**).

Solid samples of the [MnX_2_{(PhN=PPh_2_)CH_2_}] (X = Br, I) coordination compounds showed appreciable orange luminescence upon excitation with UV light, especially in the case of the bromo-complex (see for instance Figure 4). The result was unexpected, since complexes with monodentate phosphanimines having general formula [MnX_2_(NPh=PPh_3_)_2_] (X = Cl, Br, I) are known to show green photoluminescence [44]. Solutions of the two species in common organic solvents exhibited negligible luminescence, thus the investigations on the absorption and emission features were limited to the solid state. The Kubelka–Munk transformations of the reflectance spectra are observable in Figure 4, and compared to the spectrum of free (PhN=PPh_2_)CH_2_. The complexes show superimposable bands in the UV region, mainly attributable to the absorptions of the coordinated bis(phosphanimine) ligand, with K/S maximum at 280 nm. The onset falls around 380 nm, about 20 nm blue-shifted with respect to the free ligand. On the other hand, the visible range of the spectra is influenced by the nature of the coordinated halides. The superposition of unresolved, weak bands is present for wavelengths lower than 550 nm in the spectrum of [MnBr_2_{(PhN=PPh_2_)CH_2_}], attributable to the spin-forbidden ^4^G←^6^S absorptions of Mn(II) in tetrahedral field. The iodo-complex has comparatively more intense absorptions in the visible region and the wavelength range extends to about 650 nm. Cotton and Goodgame observed comparable features in the reflectance spectra of the tetrahedral phosphine oxide complexes [MnX_2_(O=PPh_3_)_2_] (X = Br, I). One of the possible explanations proposed by the authors for the reflectance spectrum of the iodo-complex was the occurrence of charge-transfer absorptions [51].

**Figure 5:**
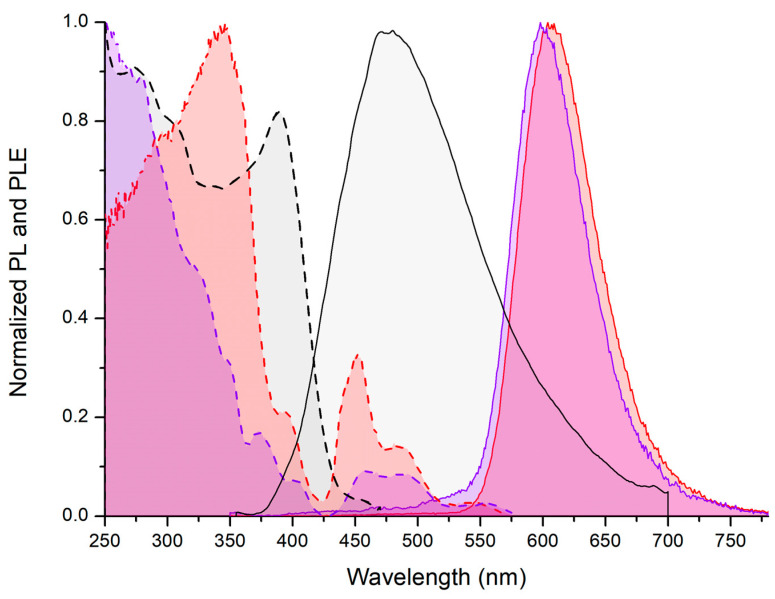
PL (solid lines, λ_excitation_ = 280 nm) and PLE spectra (dashed lines, λ_emission_ = 500–610 nm) of solid (PhN=PPh_2_)CH_2_ (black lines), [MnBr_2_{(PhN=PPh_2_)CH_2_}] (red lines), and [MnI_2_{(PhN=PPh_2_)CH_2_}] (violet lines) at 298 K.

**Figure 6:**
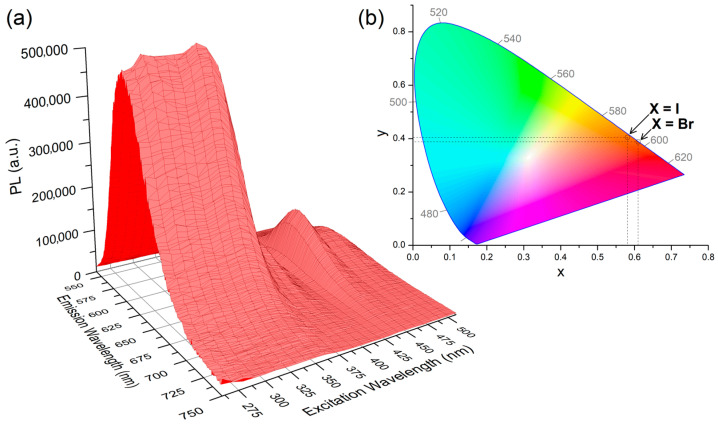
Photoluminescence 3D spectrum of solid [MnBr_2_{(PhN=PPh_2_)CH_2_}] at 298 K (**a**). CIE 1931 diagram with coordinates for [MnBr_2_{(PhN=PPh_2_)CH_2_}] and [MnI_2_{(PhN=PPh_2_)CH_2_}] (**b**).

The steady-state luminescence spectra recorded for freshly prepared samples and for crystals investigated by means of X-ray diffraction resulted superimposable, confirming that the orange photoluminescence derives from tetrahedral manganese(II) complexes. The emission (PL) spectra of the two complexes are composed by a single band centred between 598 (X = I) and 607 (X = Br) nm. The FWHM is around 1900 cm^−1^ for both the compounds. Sharp PL bands with FWHM values in this range are coherent with manganese-centred emissions [21,22]. The CIE 1931 chromaticity coordinates are x = 0.610, y = 0.388 for [MnBr_2_{(PhN=PPh_2_)CH_2_}] and x = 0.581, y = 0.405 for [MnI_2_{(PhN=PPh_2_)CH_2_}], so the emitted colours fall in the orange region of the CIE 1931 diagram with almost unitary colour purity (Figure 5 and Figure 6). The emissions are independent from the excitation wavelength and are completely different from the weak blue fluorescence centred around 475 nm of solid samples of free (PhN=PPh_2_)CH_2_, having a FWHM value around 5300 cm^−1^ and lifetime in the nanoseconds range.

The excitation (PLE) spectra of the two complexes show bands between 425 and 575 nm attributable to the ^4^G←^6^S direct excitation of Mn(II), slightly red-shifted for X = I. Using the (^4^A+^4^E)^4^G←(^6^A)^6^S transitions centred at 450 (X = Br) and 456 (X = I) nm as references, the Stokes shift is about 500 cm^−1^ lower for [MnI_2_{(PhN=PPh_2_)CH_2_}] compared to [MnBr_2_{(PhN=PPh_2_)CH_2_}], possibly because the high steric bulk of the coordinated iodides reduces the geometric variations in the complex moving from the ground to the emitting states. Another metal-centred transition is present at 394 nm for [MnBr_2_{(PhN=PPh_2_)CH_2_}] and at 403 nm for [MnI_2_{(PhN=PPh_2_)CH_2_}]. The PLE spectra differ between the two compounds at shorter wavelengths. The bromo-complex is characterized by a strong ligand-centred excitation band centred at 346 nm that hides further Mn(II)-centred excitations. The PLE profile of [MnBr_2_{(PhN=PPh_2_)CH_2_}] resembles that of free (PhN=PPh_2_)CH_2_, but the maximum is blue-shifted, as expected from the reflectance spectra. On the contrary, metal-centred transitions are clearly observable in the PLE spectrum of the iodo-derivative with local maxima at 374, 349, and 324 nm, since the phosphaminine-centred excitations in the near-UV region are absent (Figure 5).

The ligand→Mn(II) antenna effect plays an important role only for [MnBr_2_{(PhN=PPh_2_)CH_2_}]. The different behaviour of the two compounds can be tentatively justified by supposing that the excitation with near-UV light of [MnI_2_{(PhN=PPh_2_)CH_2_}] could populate excited states different from the Mn(II) ^4^G manifold, such as Mn–I charge-transfer states, with subsequent non-radiative decay. Such a hypothesis is suggested by the reflectance spectra shown in Figure 4. Another possibility is that the high spin–orbit coupling associated with the coordinated iodides [52] could favour the intersystem crossing between ligand-centred excited states subjected to fast vibrational decay.

**Figure 7:**
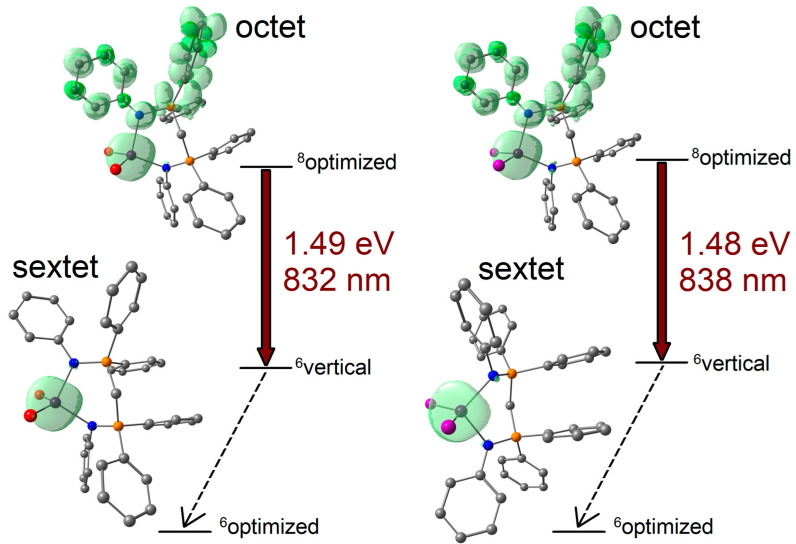
DFT-optimized structures of the [MnX_2_{(PhN=PPh_2_)CH_2_}] complexes at the sextet and octet states with spin density surfaces (green tones, surface isovalue = 0.005 a.u.). Relative energy values of the sextet-state geometries (^6^optimized), octet-state geometries (^8^optimized), and sextet states at the octet-state geometries (^6^vertical), with prediction of the ligand-centred phosphorescence. Colour map: Mn, dark violet; I, violet; Br, red; P, orange; N, blue; C, grey. Hydrogen atoms omitted for clarity.

Non-green emissions from tetrahedral manganese(II) complexes are sometimes associated with the population of ligand-centred emitting states [53,54,55,56], while in other cases, suitable ligands increase the Δ/*B* ratio and cause a shift in the Mn(II)-centred emission to longer wavelengths [27,57]. This last possibility also appears reasonable for the [MnX_2_{(PhN=PPh_2_)CH_2_}] complexes, given the sharpness of the PL bands, the presence of metal-centred excitation bands in the PLE spectra, and the excitation-independent emissions. Moreover, DFT calculations on the [MnX_2_{(PhN=PPh_2_)CH_2_}] complexes in sextet and octet states suggest that phosphorescent emissions from ligand-centred excited states should have meaningfully lower energy with respect to the observed PL bands, with predicted wavelengths in the 830–840 nm range. The DFT-optimized structures are shown in Figure 7 together with the spin density plots that evidence how the octet states are formally constituted by (PhN=PPh_2_)CH_2_ ligands in triplet state coordinated to high-spin d^5^ metal centres.

The Racah *B* parameter, estimated on the basis of the (^4^A+^4^E)^4^G←(^6^A)^6^S excitation wavelength, is between 686 (X = Br) and 677 (X = I) cm^−1^, lower that the average value obtained for related [MnX_2_(NPh=PPh_3_)_2_] derivatives, 705 cm^−1^ [44]. The nephelauxetic ratio β is around 0.74 using 923 cm^−1^ as *B* value for the free Mn^2+^ ion [58]. The ^4^G←^6^S excitation bands are red-shifted with respect to those observed for green-emitting tetrahedral manganese(II) complexes [44,46,59,60], suggesting that the coordination of (PhN=PPh_2_)CH_2_ generates a relatively strong crystal field.

**Figure 8:**
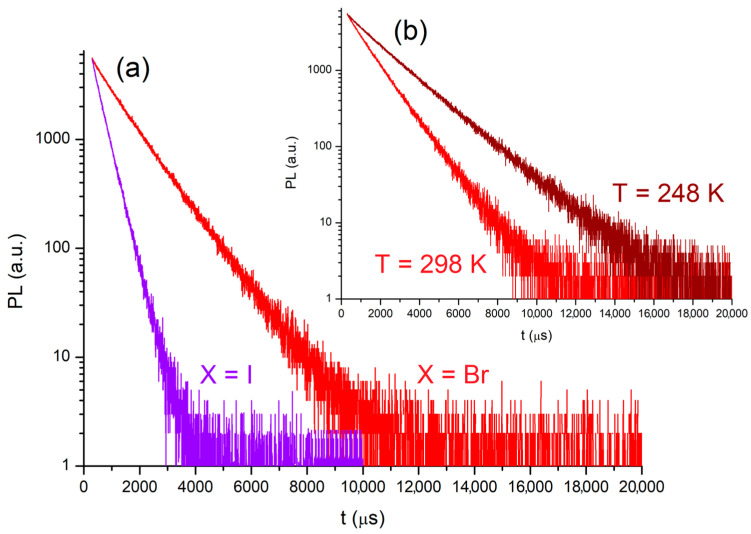
Semi-log plots of the luminescence decay curves of solid [MnBr_2_{(PhN=PPh_2_)CH_2_}] (red line, λ_excitation_ = 345 nm, λ_emission_ = 600 nm) and [MnI_2_{(PhN=PPh_2_)CH_2_}] (violet line, λ_excitation_ = 300 nm, λ_emission_ = 600 nm) at 298 K (**a**). Comparison of the luminescence decay curves of [MnBr_2_{(PhN=PPh_2_)CH_2_}] at 298 K (red line) and at 248 K (dark red line) (**b**).

The lifetimes (τ) derived from the mono-exponential fit of the luminescence decay curves recorded at 298 K (Figure 8) are equal to 1120 μs for [MnBr_2_{(PhN=PPh_2_)CH_2_}] and 373 μs for [MnI_2_{(PhN=PPh_2_)CH_2_}], much longer than the values obtained for the monodentate phosphanimine derivatives [MnX_2_(NPh=PPh_3_)_2_] (X = Br, τ = 151 μs; X = I, τ = 55 μs) [44]. The τ value of [MnBr_2_{(PhN=PPh_2_)CH_2_}] is comparable to that measured for the tetrahedral bromo-complex [MnBr_2_(DBFDPO)], having the bidentate phosphine oxide 4,6-bis(diphenylphosphoryl)dibenzofuran (DBFDPO) in the coordination sphere [24,27]. The lower τ value of the iodo-complex is almost in part attributable to the acceleration of the radiative decay, ascribed to the increased degree of spin–orbit coupling [52].

Given the wider excitation range, [MnBr_2_{(PhN=PPh_2_)CH_2_}] was subjected to further investigations. The relative energy value computed for the DFT-optimized octet state is around 21,900 cm^−1^, while the onset of the PL band is at about 18,500 cm^−1^. The observed antenna effect is thus supported by the computational calculations. The photoluminescence quantum yield (Φ) at room temperature is about 6%, probably because of competitive vibrational decay. On the basis of the equation Φ = k_r_(k_r_ + k_nr_)^−1^ = τk_r_, the radiative (k_r_) and non-radiative (k_nr_) rate constant are estimated around 5.4∙10^1^ and 8.4∙10^2^ s^−1^, respectively [61]. The radiative decay rate is about one order of magnitude slower than that of [MnBr_2_(DBFDPO)] (Φ = 81.4%, τ = 1.0 ms) [24]. Lowering the temperature to 248 K did not cause any appreciable variation in the PL and PLE spectra of [MnBr_2_{(PhN=PPh_2_)CH_2_}], but the lifetime of the emission noticeably increased to 1878 μs, a result in line with the expected decrease in k_nr_ (Figure 8).

## 3. Experimental Section

### 3.1. Materials and Methods

Anhydrous manganese(II) halides and other inorganic and organic reactants were purchased from Merck (Darmstadt, Germany). Solvents were purified prior to use according to established methods [62]. Deuterated chloroform (Euriso-Top, Saarbrücken, Germany) was used as received. Phenyl azide was synthesized from phenylhydrazine under a fume hood following a reported procedure. The distillation step was omitted, and the compound was purified by silica gel filtration using pentane as eluent [63]. *Safety note: Organic azides are potentially explosive compounds. All manipulations were performed using limited quantities of reactants, with careful temperature control throughout the synthetic steps.* (PhN=PPh_2_)CH_2_ was synthesized from phenyl azide and bis(diphenylphosphino)methane according to methods in the literature [64]. The moisture-sensitive compounds were prepared and stored in a MBraun MB10 glove box (Garching bei München, Germany) filled with nitrogen and equipped for both organic and inorganic syntheses.

### 3.2. Characterizations

Elemental analyses for carbon, hydrogen, and nitrogen were performed using an Elementar (Langenselbold, Germany) Unicube microanalyzer. Halide contents were determined by Mohr’s method [65]. Magnetic susceptibility measurements were carried out on solid samples at 298 K under a magnetic field strength of 3.5 kGauss using an MK1 magnetic susceptibility balance (Sherwood Scientific Ltd., Cambridge, UK). The measured values were corrected for diamagnetic contributions using tabulated Pascal’s constants [66]. FT-IR spectra of the species dispersed in KBr (spectroscopy grade, Merck) were recorded in the 4000–450 cm^−1^ range using a Perkin-Elmer (Shelton, CT, USA) Spectrum One spectrophotometer. Samples were prepared under N_2_ atmosphere in a glove box. ^31^P{^1^H} NMR spectra were recorded on a Bruker (Billerica, MA, USA) Avance 400 spectrometer operating at 400.13 MHz for ^1^H resonance. ^31^P chemical shifts are reported relative to external 85% H_3_PO_4_ in water. Melting points were registered using a FALC (Treviglio, Italy) 360 D instrument equipped with a camera. Thermogravimetric analyses (TGA) were carried out under N_2_ atmosphere with a Perkin-Elmer TGA 4000 instrument.

### 3.3. Synthesis of [MnX_2_{(PhN=PPh_2_)CH_2_}] (X = Br, I)

Anhydrous MnX_2_ (0.5 mmol; X = Br, 0.107 g; X = I, 0.154 g) was dissolved in 25 mL of acetonitrile. A stoichiometric amount of (PhN=PPh_2_)CH_2_ (0.5 mmol, 0.283 g) was added and the reaction mixture was kept stirring at room temperature for 12 h. The solvent was then removed by evaporation at reduced pressure and dichloromethane (25 mL) was added. The solution was purified by centrifugation. Diethyl ether (10 mL) was added after evaporation of the solvent, and the solid that separated was collected by filtration, washed with 5 mL of diethyl ether, and dried under vacuum. Yield: 0.301 g (77%) for X = Br; 0.302 g (69%) for X = I.

*Characterization of [MnBr_2_{(PhN=PPh_2_)CH_2_}]*. Anal. calcd. for C_37_H_32_Br_2_MnN_2_P_2_ (781.36 g mol^−1^, %): C, 56.87; H, 4.13; N, 3.59; Br, 20.45. Found (%): 56.65; H, 4.15; N, 3.57; Br, 20.36. IR (KBr, cm^−1^): 1591, 1489 (ν_CC_); 1250, 1237 (ν_PN_). χ_M_^corr^ (293 K, cgsu): 1.50 × 10^−2^ (μ = 5.9 BM). ^31^P{^1^H} NMR (CDCl_3_, 300 K): δ 26.6 (s, FWHM = 50 Hz). M.p. (°C): 130 (mass loss > 230 °C).
*Characterization of [MnI_2_{(PhN=PPh_2_)CH_2_}]*. Anal. calcd. for C_37_H_32_I_2_MnN_2_P_2_ (875.36 g mol^−1^, %): C, 50.77; H, 3.68; N, 3.20; I, 28.99. Found (%): 50.58; H, 3.70; N, 3.18; I, 28.88. IR (KBr, cm^−1^): 1591, 1489 (ν_CC_); 1227 (ν_PN_). χ_M_^corr^ (293 K, cgsu): 1.47 × 10^−2^ (μ = 5.9 BM). ^31^P{^1^H} NMR (CDCl_3_, 300 K): δ 26.6 (s, FWHM = 35 Hz). M.p. (°C): 132 (mass loss > 230 °C).

### 3.4. Single-Crystal X-Ray Structure Determinations

Crystallographic data were collected at CACTI (Universidade de Vigo) at 100 K using a Bruker D8 Venture diffractometer with a Photon II CMOS detector and Mo-Kα radiation (λ = 0.71073 Å) generated by an Incoatec (Geesthacht, Germany) high-brillance microfocus source equipped with Incoatec Helios multilayer optics. The software APEX5 version 2023.9-4 [67] was used for collecting frames of data, indexing reflections, and determination of lattice parameters, SAINT version 8.40B [68] for integration of intensity of reflections, and SADABS version 2016/2 [69] for scaling and empirical absorption correction. Further crystallographic treatment was performed with the Oscail programme v.4.7.1 [70]. The structures of the compounds were solved by using the SHELXT version 2018/2 programme [71] and refined by a full-matrix least-squares method based on *F*^2^ using the SHELXL version 2019/2 programme [72]. Non-hydrogen atoms were refined with anisotropic displacement parameters. Hydrogen atoms were included in idealized positions and refined with isotropic displacement parameters. The PLATON programme (version: 80125) [73] was used to obtain some geometrical parameters from the cif files. CCDC 2499368 and 2499369 contain the supplementary crystallographic data. These data can be obtained free of charge from the Cambridge Crystallographic Data Centre via www.ccdc.cam.ac.uk/structures (accessed on 18 November 2025). Other details about crystal data and structural refinement are given in Table S3. The optimization of the positions of the hydrogen atoms in the crystals by means of periodic DFT calculations is described in the Supplementary Materials, Table S4.

### 3.5. Reflectance and Photoluminescence Measurements

Reflectance and luminescence measurements were carried out on powder samples placed in air-tight quartz sample holders filled in a glove box to avoid interactions of the compounds with moisture. Reflectance spectra in the 210–850 nm range were collected at room temperature with a Jasco (Tokyo, Japan) V-770 spectrophotometer equipped with a 150 mm Jasco ILN-925 integrating sphere. Spectroscopy-grade polytetrafluoroethylene powder was used as reference. Photoluminescence emission (PL) and excitation (PLE) spectra as well as lifetime decay curves were registered using an Edinburgh Instruments (Livingston, Scotland, UK) FS5 spectrofluorometer with a Peltier-based temperature controller. Suitable long-pass filters were placed in front of the acquisition systems. The excitation and emission spectra were corrected for the instrumental functions. Time-resolved analyses were performed in multi-channel scaling mode (MCS) using an integrated pulsed xenon lamp and in time-correlated single-photon counting (TCSPC) with an Edinburgh Instruments EPLED pulsed led centred at 365 nm. The room temperature photoluminescence quantum yield (Φ) of [MnBr_2_{(PhN=PPh_2_)CH_2_}] at the solid state was measured with an OceanOptics (Orlando, FL, USA) HR4000CG detector fibre-coupled to an integrating sphere connected to an OceanOptics UV LED continuous source (λ_max_ = 365 nm). The value reported is the average of three measurements.

*Photoluminescence data for [MnBr_2_{(PhN=PPh_2_)CH_2_}]*. PL (solid, λ_excitation_ = 280 nm, 298 K, nm): 607 (FWHM = 1900 cm^−1^) ^4^T_1_(^4^G)→^6^A(^6^S). CIE 1931: x = 0.610; y = 0.388. PLE (solid, λ_emission_ = 610 nm, 298 K, nm): <385 (max 346) ligand-centred and metal-centred; 390–575 (local max 394, 450, 487, 543) metal-centred. τ (solid, λ_excitation_ = 345 nm, λ_emission_ = 600 nm, μs): 1120 (T = 298 K), 1878 (T = 248 K). Φ (solid, λ_excitation_ = 360 nm): 6%.
*Photoluminescence data for [MnI_2_{(PhN=PPh_2_)CH_2_}]*. PL (solid, λ_excitation_ = 280 nm, 298 K, nm): 598 (FWHM = 1900 cm^−1^) ^4^T_1_(^4^G)→^6^A(^6^S). CIE 1931: x = 0.581; y = 0.405. PLE (solid, λ_emission_ = 610 nm, 298 K, nm): <360 ligand-centred and metal-centred; 365–575 (local max 374, 403, 456, 491, 550) metal-centred. τ (solid, λ_excitation_ = 300 nm, λ_emission_ = 600 nm, μs): 373 (T = 298 K).

### 3.6. Computational Simulations of Single Molecules

The geometric optimizations of the complexes were carried out using the hybrid *meta*-GGA DFT functional TPSS0, with 25% HF exchange [74], in combination with Ahlrichs’ def2-TZVP basis set and relativistic ECP for iodine [75,76,77]. The D4 method for London dispersion correction was added to the calculations [78]. The “unrestricted” approach was used, and the absence of meaningful spin contamination was verified by comparing the computed <*S*^2^> values with the theoretical ones. The stationary points were characterized as true minima by means of IR simulations, carried out with the harmonic approximation [79]. Calculations were carried out using ORCA version 5.0.3 [80,81] and the results were elaborated with Multiwfn version 3.8 [82,83]. Cartesian coordinates of the DFT-optimized structures are collected in Table S5.

## 4. Conclusions

Manganese is an inexpensive, non-toxic, and earth-abundant element, and these features currently drive the development of manganese-based compounds of interest for advanced technologies. We reported that the formal replacement of bidentate phosphine oxides with electronically comparable bis(phosphanimine) ligands in the coordination sphere of manganese(II) can yield luminescent coordination compounds with unexpected emission features, despite the tetrahedral coordination geometry of the final products. Polydentate phosphanimines thus appear to be promising candidates for the synthesis of new sustainable luminescent coordination compounds. Further studies are necessary to improve the luminescence performances, particularly the quantum yield. Moreover, the preparation of materials for optics and lighting requires investigating the embedding of phosphanimine-based manganese(II) complexes in suitable matrices.

## Data Availability

Data are contained within the article and Supplementary Materials.

## Supplementary Materials

The following supporting information can be downloaded at https://www.mdpi.com/article/10.3390/molecules31010161/s1. Figure S1: ^1^H NMR spectra of [MnX_2_{(PhN=PPh_2_)CH_2_}]; Figure S2: TGA curves of (PhN=PPh_2_)CH_2_, [MnBr_2_{(PhN=PPh_2_)CH_2_}] and [MnI_2_{(PhN=PPh_2_)CH_2_}]; Figure S3: Superposition of the structures of the [MnX_2_{(PhN=PPh_2_)CH_2_}] complexes; Table S1: Descriptors for the four-coordinate geometry for [MnX_2_{(PhN=PPh_2_)CH_2_}]; Figure S4: Torsion angles in the six-membered metallacycles of [MnX_2_{(PhN=PPh_2_)CH_2_}]; Figure S5: Supramolecular interactions in the crystal structure of [MnBr_2_{(PhN=PPh_2_)CH_2_}]; Table S2: Parameters for the intermolecular interactions in the crystal structures of [MnX_2_{(PhN=PPh_2_)CH_2_}]; Table S3: Crystal data and structure refinement for [MnX_2_{(PhN=PPh_2_)CH_2_}]; Table S4: Stationary points obtained after periodic DFT calculations; Table S5: Cartesian coordinates of the DFT-optimized geometries of [MnX_2_{(PhN=PPh_2_)CH_2_}]. Refs. [84,85,86,87,88,89,90,91,92,93] are cited in Supplementary Materials.
