# Supplementary material for: Unexpected Orange Photoluminescence from Tetrahedral Manganese(II) Halide Complexes with Bidentate Phosphanimines
Source: Molecules. 2026 Jan 1;31(1):161. doi: 10.3390/molecules31010161 (PMC12787953; doi:10.3390/molecules31010161)

checkCIF/PLATON report

Structure factors have been supplied for datablock(s) 32118102_0t_a

THIS REPORT IS FOR GUIDANCE ONLY. IF USED AS PART OF A REVIEW PROCEDURE FOR PUBLICATION, IT SHOULD NOT REPLACE THE EXPERTISE OF AN EXPERIENCED CRYSTALLOGRAPHIC REFEREE.

No syntax errors found. CIF dictionary Interpreting this report

Datablock: 32118102_0t_a

```
Bond precision:   C-C = 0.0046 A          Wavelength=0.71073

Cell:            a=19.5117(14)     b=12.3042(8)       c=16.8889(13)
                 alpha=90          beta=114.209(3)    gamma=90
Temperature:     100 K
```

	Calculated	Reported
Volume	3698.0(5)	3698.0(5)
Space group	P 21/c	P 21/c
Hall group	-P 2ybc	-P 2ybc
Moiety formula	C37 H32 Br2 Mn N2 P2, C H2 Cl2	C37 H32 Br2 Mn N2 P2, C H2 Cl2
Sum formula	C38 H34 Br2 Cl2 Mn N2 P2	C38 H34 Br2 Cl2 Mn N2 P2
Mr	866.25	866.27
Dx,g cm-3	1.556	1.556
Z	4	4
Mu (mm-1)	2.779	2.779
F000	1740.0	1740.0
F000'	1741.50	
h,k,lmax	25,16,21	25,15,21
Nref	8510	8472
Tmin,Tmax	0.649,0.826	0.555,0.714
Tmin'	0.636	

Correction method= # Reported T Limits: Tmin=0.555 Tmax=0.714
AbsCorr = MULTI-SCAN

```
Data completeness= 0.996            Theta(max)= 27.543

R(reflections)= 0.0382( 7198)                       wR2(reflections)=
                                                    0.1008( 8472)
S = 1.054                   Npar= 424
```

The following ALERTS were generated. Each ALERT has the format
test-name_ALERT_alert-type_alert-level.
Click on the hyperlinks for more details of the test.

Alert level C

```
PLAT911_ALERT_3_C Missing FCF Refl Between Thmin & STh/L=    0.600          9 Report
               -1  1  1,  -2 11  8,  -5  1 12,  -4  1 13,  -5  2 14,  -4  1 14,
               -5  3 15,  -6  2 16,  -7  1 17,
```

Alert level G

```
PLAT083_ALERT_2_G SHELXL Second Parameter in WGHT  Unusually Large       8.45 Why ?
PLAT232_ALERT_2_G Hirshfeld Test Diff (M-X)  Br1      --Mn       .        9.5 s.u.
PLAT232_ALERT_2_G Hirshfeld Test Diff (M-X)  Br2      --Mn       .       10.3 s.u.
PLAT720_ALERT_4_G Number of Unusual/Non-Standard Labels .........           2 Note
              H1S1    H1S2
PLAT794_ALERT_5_G Tentative Bond Valency for Mn        (II)      .       2.33 Info
PLAT910_ALERT_3_G Missing FCF Reflection(s) Below Theta(Min)[Deg]=       2.01 Note
                1  0  0,
PLAT912_ALERT_4_G Missing # of FCF Reflections Above STh/L=  0.600         27 Note
PLAT933_ALERT_2_G Number of HKL-OMIT Records in Embedded .res File          3 Note
               -1  1  1,  -5  1 12,  -2 11  8,
PLAT969_ALERT_5_G The 'Henn et al.' R-Factor-gap value ..........       2.791 Note
              Predicted wR2: Based on SigI**2  3.61 or SHELX Weight  9.56
PLAT978_ALERT_2_G Number C-C Bonds with Positive Residual Density.          2 Info
```

---

0 **ALERT level A** = Most likely a serious problem - resolve or explain
0 **ALERT level B** = A potentially serious problem, consider carefully
1 **ALERT level C** = Check. Ensure it is not caused by an omission or oversight
10 **ALERT level G** = General information/check it is not something unexpected

0 ALERT type 1 CIF construction/syntax error, inconsistent or missing data
5 ALERT type 2 Indicator that the structure model may be wrong or deficient
2 ALERT type 3 Indicator that the structure quality may be low
2 ALERT type 4 Improvement, methodology, query or suggestion
2 ALERT type 5 Informative message, check

It is advisable to attempt to resolve as many as possible of the alerts in all categories. Often the minor alerts point to easily fixed oversights, errors and omissions in your CIF or refinement strategy, so attention to these fine details can be worthwhile. It is up to the individual to critically assess their own results and, if necessary, seek expert advice.

PLATON version of 26/09/2025; check.def file version of 20/09/2025

duplicate check

No duplication found

Datablock 32118102_0t_a - ellipsoid plot

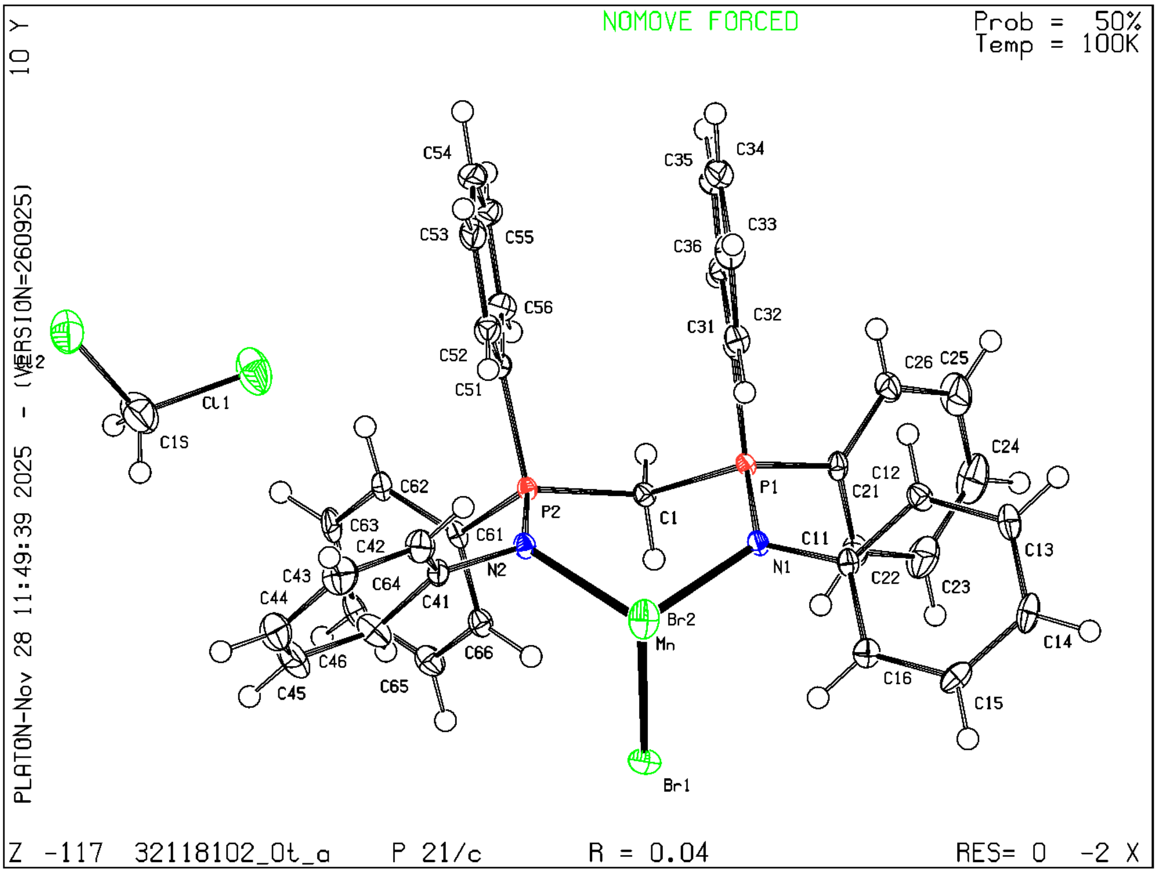

Supplement: Supplementary file 1 [file molecules-31-00161-s001.zip › bromo-complex-checkcif.pdf]
