# Supplementary material for: Unexpected Orange Photoluminescence from Tetrahedral Manganese(II) Halide Complexes with Bidentate Phosphanimines
Source: Molecules. 2026 Jan 1;31(1):161. doi: 10.3390/molecules31010161 (PMC12787953; doi:10.3390/molecules31010161)
Supplement: Supplementary file 1 [file molecules-31-00161-s001.zip › Supplementary-revised.pdf]

Unexpected Orange Photoluminescence from Tetrahedral Manganese(II) Halide Complexes with Bidentate Phosphanimines

Domenico Piccolo [1,2], Jesús Castro [3], Valentina Beghetto [1,4], Daniele Rosa-Gastaldo [2] and Marco Bortoluzzi [1,4,*]

1. Dipartimento di Scienze Molecolari e Nanosistemi, Università Ca' Foscari Venezia, 30172 Mestre, Italy; domenico.piccolo@unive.it, beghetto@unive.it
2. Dipartimento di Scienze Chimiche, Università di Padova, Via Marzolo 1, 35131 Padova, Italy; daniele.rosagastaldo@unipd.it
3. Departamento de Química Inorgánica, Facultade de Química, Universidade de Vigo, Edificio de Ciencias Experimentais, 36310 Vigo, Spain; jesusc@uvigo.gal
4. CIRCC (Consorzio Universitario Reattività Chimica e Catalisi), Via Celso Ulpiani 27, 70126 Bari, Italy.

* Correspondence: markos@unive.it; Tel.: +39 0412348561

Supplementary Materials

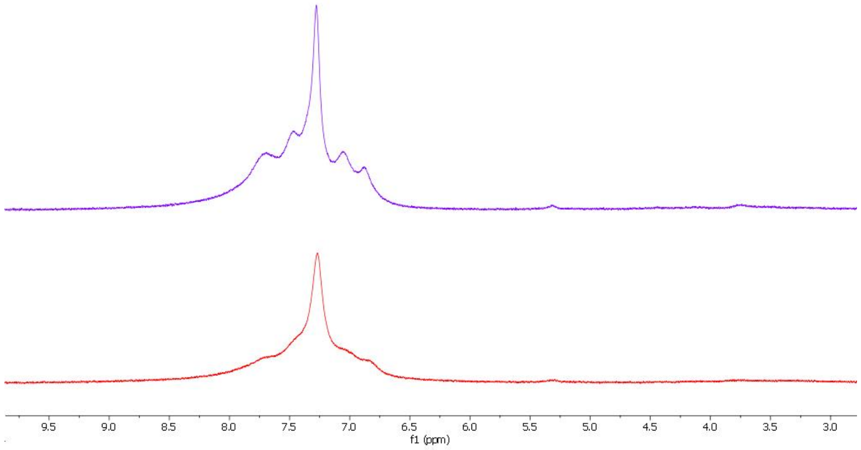

Figure S1. ^{1}H NMR spectra of $[MnX_2\{(PhN{=}PPh_2)CH_2\}]$ (X = Br, red line; X = I, violet line; $CDCl_3$, 300 K).

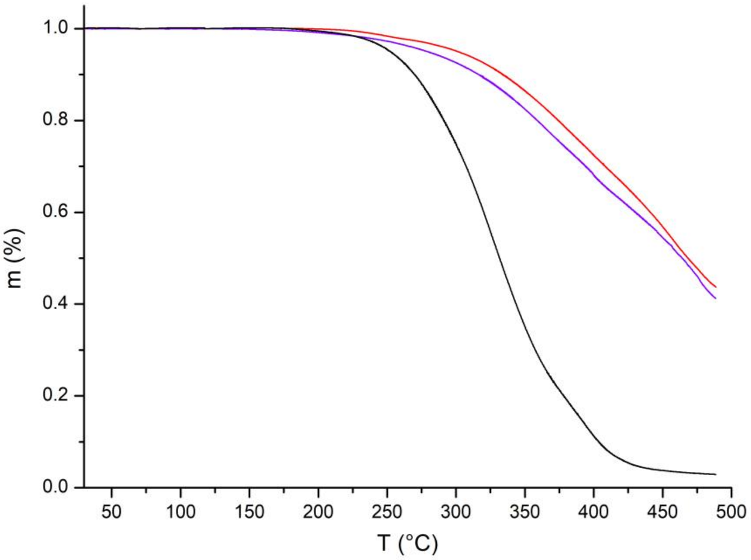

Figure S2. TGA curves of $(PhN{=}PPh_2)CH_2$ (black line), $[MnBr_2\{(PhN{=}PPh_2)CH_2\}]$ (red line) and $[MnI_2\{(PhN{=}PPh_2)CH_2\}]$ (violet line).

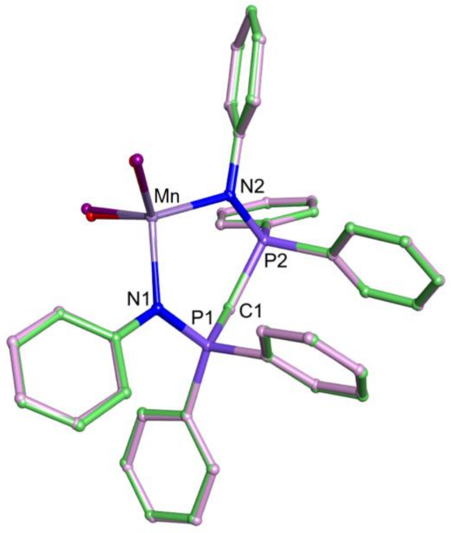

Figure S3. Superposition of the structures of the $[MnX_2\{(PhN{=}PPh_2)CH_2\}]$ complexes (Br red, I violet, C atoms in $[MnBr_2\{(PhN{=}PPh_2)CH_2\}]$ green, C atoms in $[MnI_2\{(PhN{=}PPh_2)CH_2\}]$ plum).

Table S1. Descriptors for the four-coordinate geometry for $[MnX_2\{(PhN{=}PPh_2)CH_2\}]$ (output of the Continuous Shape Measures calculations [1,2] and values of τ_4 and τ'_4 [3,4] for the complexes).

	**SP** [a]	**TET** [b]	**SS** [c]	**vTBPY** [d]	τ_4 [e]	τ'_4 [e]
**X = Br**	28.598	1.834	4.848	2.902	0.87	0.82
**X = I**	29.486	2.208	5.297	3.190	0.89	0.85

[a] SP, Square planar. [b] TET, Tetrahedron. [c] SS, Seesaw. [d] vTBPY, Vacant trigonal bipyramid. [e] τ_4 and τ'_4 are classical descriptors for four coordination; extreme forms: 0.00 for SP and 1.00 for TET.

[1] Álvarez, S.; Alemany, P.; Casanova, D.; Cirera, J.; Llunell, M.; Avnir, D. Shape maps and polyhedral interconversion paths in transition metal chemistry. *Coord. Chem. Rev.* **2005**, *249*, 1693–1708. [2] Cirera, J.; Alemany, P.; Álvarez, S. Mapping the Stereochemistry and Symmetry of Tetracoordinate Transition-Metal Complexes. *Chem. Eur. J.* **2004**, *10*, 190–207. [3] Yang, L.; Powell, D.R.; Houser, R.P. Structural variation in copper(I) complexes with pyridylmethylamide ligands: structural analysis with a new four-coordinate geometry index, τ_4. *Dalton Trans.* **2007**, 955–964. [4] Okuniewski, A.; Rosiak, D.; Chojnacki, J.; Becker, B. Coordination polymers and molecular structures among complexes of mercury(II) halides with selected 1-benzoylthioureas. *Polyhedron* **2015**, *90*, 47–57.

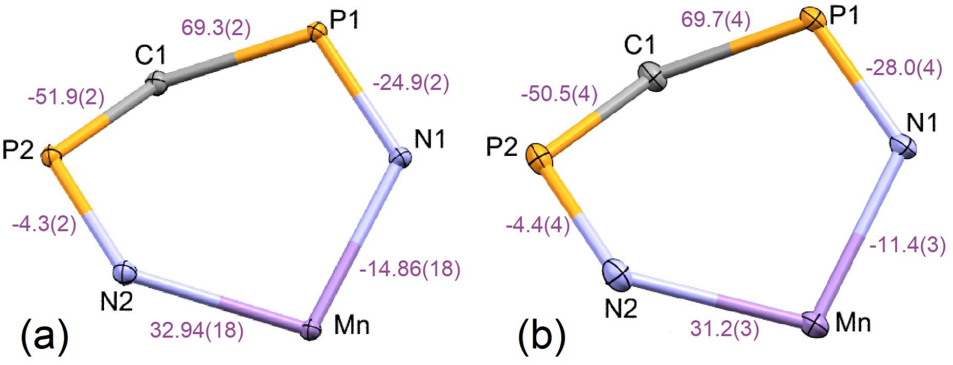

Figure S4. Torsion angles in the six membered metallacycles of $[MnX_2\{(PhN{=}PPh_2)CH_2\}]$. **(a)** X = Br; puckering parameters: Q 0.731(2) Å, θ 98.66(16)°, φ 343.46(16)°; **(b)** X = I; puckering parameters: Q 0.713(4) Å, θ, 100.5(3)°, φ 341.2(3)°.

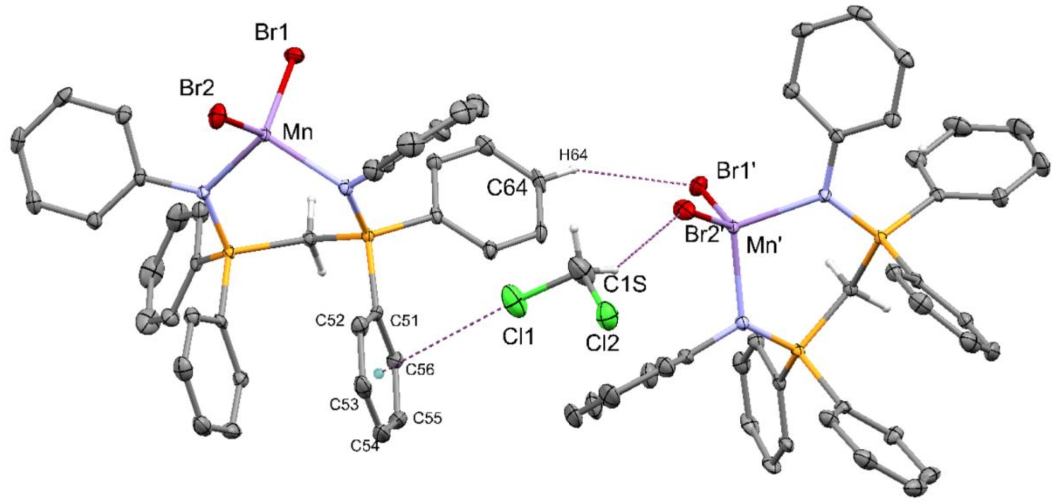

Figure S5. Supramolecular interactions in the crystal structure of $[MnBr_2\{(PhN{=}PPh_2)CH_2\}]$.

Table S2. Parameters for the intermolecular interactions in the crystal structures of $[MnX_2\{(PhN{=}PPh_2)CH_2\}]$ (values after periodic DFT optimization of the hydrogen atoms in square brackets).

**Interaction** [a]	**X**	**d(D-H), Å**	**d(H...A), Å**	**d(D...A), Å**	**<(DHA), °**
C(1S)-H(1S2)...X(2i)	Br	0.99 [1.10]	2.91 [2.83]	3.822(4)	153.2 [150]
	I	0.99 [1.10]	2.92 [2.82]	3.900(10)	171.0 [168]
C(64)-H(64)...X(1i)	Br	0.95 [1.09]	2.93 [2.76]	3.803(3)	153.4 [152]
	I	0.95 [1.10]	3.04 [2.90]	3.902(5)	152.0 [152]
**Interaction** [b]	**X**	**d(Cl...Ct), Å**	**d(X-Perp), Å** [c]	**<(C-Cl..Ct), °**	**<(C-Cl,π), °** [d]
C(1S)-Cl(1)...Ct	Br	3.6756(16)	3.454	176.13(16)	73.81
	I	3.637(3)	3.432	177.4(4)	d68.34

[a] Symmetry operation used: i: x, 3/2-y, z-1/2. [b] Ct: centroid of the phenyl ring labelled C51 to C56. [c] X-Perp: perpendicular distance of Cl atom to ring plane. [d] <(C-Cl,π): angle of the C-Cl bond with the π-plane.

Table S3. Crystal data and structure refinement for $[MnX_2\{(PhN{=}PPh_2)CH_2\}]$.

	**$[MnBr_2\{(PhN{=}PPh_2)CH_2\}]$**	**$[MnI_2\{(PhN{=}PPh_2)CH_2\}]$**
CCDC number	2499368	2499369
Empirical formula	$C_{38}\ H_{34}\ Br_2\ Cl_2\ Mn\ N_2\ P_2$	$C_{38}\ H_{34}\ Cl_2\ I_2\ Mn\ N_2\ P_2$
Moiety formula	$C_{37}\ H_{32}\ Br_2\ Mn\ N_2\ P_2$, $C\ H_2\ Cl_2$	$C_{37}\ H_{32}\ I_2\ Mn\ N_2\ P_2$, $C\ H_2\ Cl_2$
Formula weight	866.27	960.25
Temperature	100(2) K	100(2) K
Wavelength	0.71073 Å	0.71073 Å
Crystal system	Monoclinic	Monoclinic
Space group	$P2_1/c$	$P2_1/c$
Unit cell dimensions	a = 19.5117(14) Å<br>b = 12.3042(8) Å<br>c = 16.8889(13) Å<br>β = 114.209(3)°	a = 19.8111(9) Å<br>b = 12.2760(6) Å<br>c = 17.3269(8) Å<br>β = 115.186(2)°
Volume	3698.0(5) $Å^3$	3813.3(3) $Å^3$
Z	4	4
Density (calculated)	1.556 Mg/m^3	1.673 Mg/m^3
Absorption coefficient	2.779 mm^{-1}	2.219 mm^{-1}
F(000)	1740	1884
Crystal size	0.159 x 0.134 x 0.069 mm	0.115 x 0.108 x 0.075 mm
Theta range for data collection	2.012 to 27.543°	2.011 to 28.311°
Index ranges	$-25 \leq h \leq 25$<br>$-15 \leq k \leq 15$<br>$-21 \leq l \leq 21$	$-26 \leq h \leq 26$<br>$-16 \leq k \leq 16$<br>$-23 \leq l \leq 19$
Reflections collected	93912	55982
Independent reflections	8472 [R_{int} = 0.0733, $R\sigma$ = 0.0334]	9475 [R_{int} = 0.0565, $R\sigma$ = 0.0401]
Reflections observed (>2σ)	7198	7571
Data Completeness	0.996	0.998
Absorption correction	Semi-empirical from equivalents	Semi-empirical from equivalents
Max. and min. transmission	0.7144 and 0.5548	0.7457 and 0.5804
Refinement method	Full-matrix least-squares on F^2	Full-matrix least-squares on F^2
Data / restraints / parameters	8472 / 0 / 424	9475 / 0 / 424
Goodness-of-fit on F^2	1.054	1.059
Final R indices [I>2σ(I)]	R_1 = 0.0382<br>wR_2 = 0.0944	R_1 = 0.0519<br>wR_2 = 0.1330
R indices (all data)	R_1 = 0.0489<br>wR_2 = 0.1008	R_1 = 0.0696<br>wR_2 = 0.1453
Largest diff. peak and hole	1.498 and -1.001 e.$Å^{-3}$	1.294 and -2.675 e.$Å^{-3}$

Table S4. Stationary points obtained after periodic DFT calculations [a] (cif format).

**$[MnBr_2\{(PhN{=}PPh_2)CH_2\}]\cdot CH_2Cl_2$**	**$[MnI_2\{(PhN{=}PPh_2)CH_2\}]\cdot CH_2Cl_2$**
data_MnBr2dppmNPh2-DCM-PBESOL	data_MnI2dppmNPh2-DCM-PBESOL
_symmetry_cell_setting monoclinic	_symmetry_cell_setting monoclinic
_symmetry_space_group_name_H-M 'P 21/c'	_symmetry_space_group_name_H-M 'P 21/c'
_symmetry_Int_Tables_number 14	_symmetry_Int_Tables_number 14
loop_	loop_
_symmetry_equiv_pos_site_id	_symmetry_equiv_pos_site_id
_symmetry_equiv_pos_as_xyz	_symmetry_equiv_pos_as_xyz
1 x,y,z	1 x,y,z
2 -x,1/2+y,1/2-z	2 -x,1/2+y,1/2-z
3 -x,-y,-z	3 -x,-y,-z
4 x,1/2-y,1/2+z	4 x,1/2-y,1/2+z
_cell_length_a 19.5117000	_cell_length_a 19.8111000
_cell_length_b 12.3042000	_cell_length_b 12.2760000
_cell_length_c 16.8889000	_cell_length_c 17.3269000
_cell_angle_alpha 90.0000000	_cell_angle_alpha 90.0000000

_cell_angle_beta 114.2090000	_cell_angle_beta 115.1860000
_cell_angle_gamma 90.0000000	_cell_angle_gamma 90.0000000
_cell_volume 3698.04	_cell_volume 3813.31
loop_	loop_
_atom_site_label	_atom_site_label
_atom_site_type_symbol	_atom_site_type_symbol
_atom_site_fract_x	_atom_site_fract_x
_atom_site_fract_y	_atom_site_fract_y
_atom_site_fract_z	_atom_site_fract_z
H H 0.1778194532 0.3758753409 0.4596369931	H H 0.1247055367 0.4219473994 0.5075002406
H H 0.1225267319 0.4288311155 0.5119963965	H H 0.1810838062 0.3672342322 0.4604955474
H H 0.2183979174 0.0996647786 0.6911939038	H H 0.2172804326 0.0952121862 0.6876825221
H H 0.1660792128 -0.0381524142 0.7569523480	H H 0.1675685764 -0.0403762836 0.7551749167
H H 0.0791610324 0.0173892765 0.8250832938	H H 0.0860853462 0.0183220237 0.8243535944
H H 0.0525164940 0.2128625399 0.8335768382	H H 0.0591535101 0.2146592593 0.8297197722
H H 0.1031830009 0.3521011844 0.7654975665	H H 0.1058169662 0.3512274359 0.7582646797
H H 0.0398354938 0.2893566426 0.5184989785	H H 0.0423395396 0.2752875429 0.5144989669
H H -0.0581235361 0.1665285922 0.4195688726	H H -0.0508489893 0.1480984327 0.4166711098
H H -0.0216562296 -0.0044293203 0.3682547941	H H -0.0113693826 -0.0183773078 0.3682564432
H H 0.1126922178 -0.0515372122 0.4153170136	H H 0.1233563587 -0.0608546750 0.4192277069
H H 0.2112770142 0.0697817983 0.5133556469	H H 0.2181416129 0.0646511339 0.5174613896
H H 0.3218123331 0.2568321293 0.7553270560	H H 0.3201008334 0.2533655321 0.7547696498
H H 0.4526072439 0.1872549834 0.7996207934	H H 0.4511979047 0.1861378824 0.8052173984
H H 0.4957454971 0.1277146623 0.6875047179	H H 0.4974153958 0.1288171704 0.6986542842
H H 0.4098106945 0.1413284265 0.5302491842	H H 0.4161664089 0.1426801159 0.5441413716
H H 0.2812003949 0.2085132088 0.4859989793	H H 0.2869762053 0.2069067784 0.4937164370
H H 0.3681240780 0.5966282660 0.7932613005	H H 0.3734212118 0.5945800505 0.7838895506
H H 0.4174785231 0.7743699320 0.8607902000	H H 0.4186809506 0.7736474021 0.8494885904
H H 0.3546276160 0.9467314854 0.7873809317	H H 0.3511426775 0.9437710315 0.7801204916
H H 0.2424155546 0.9348723517 0.6460605355	H H 0.2360912084 0.9284213985 0.6436447521
H H 0.1925856541 0.7570740475 0.5817497914	H H 0.1876289406 0.7486515504 0.5818546873
H H 0.3910679974 0.5056874441 0.6821991858	H H 0.3923859592 0.5018963370 0.6798991259
H H 0.5099242161 0.4412502240 0.6751823231	H H 0.5103401826 0.4434964696 0.6738179736
H H 0.5093260795 0.3820518051 0.5339143036	H H 0.5095504824 0.3842490222 0.5366289141
H H 0.3913262802 0.3855605602 0.4010552444	H H 0.3918878344 0.3823372312 0.4055596872
H H 0.2735928912 0.4492678841 0.4066721925	H H 0.2743296598 0.4444185338 0.4091498629
H H 0.2838572390 0.6514886021 0.4318983184	H H 0.2832103060 0.6487090089 0.4335067539
H H 0.2105859952 0.7772595422 0.3133688727	H H 0.2084778262 0.7749223203 0.3169447437
H H 0.0767774751 0.8133122347 0.2791450487	H H 0.0763669149 0.8084743306 0.2836299948
H H 0.0158138421 0.7262220380 0.3674536232	H H 0.0178593935 0.7194320380 0.3686685538
H H 0.0883764584 0.6018963209 0.4877644323	H H 0.0906510971 0.5931207682 0.4854947979
C C 0.1801800000 0.4029000000 0.5232500000	C C 0.1824000000 0.3959000000 0.5216000000
C C 0.1620700000 0.2351000000 0.7204700000	C C 0.1623000000 0.2326000000 0.7150000000
C C 0.1808600000 0.1253000000 0.7212300000	C C 0.1811000000 0.1219000000 0.7172000000
C C 0.1517200000 0.0475000000 0.7588600000	C C 0.1537000000 0.0457000000 0.7560000000
C C 0.1044600000 0.0782000000 0.7981000000	C C 0.1091000000 0.0784000000 0.7955000000
C C 0.0888100000 0.1877000000 0.8016800000	C C 0.0933000000 0.1878000000 0.7975000000
C C 0.1172400000 0.2661000000 0.7639500000	C C 0.1196000000 0.2649000000 0.7575000000
C C 0.1317600000 0.1871000000 0.5228200000	C C 0.1366000000 0.1785000000 0.5227000000
C C 0.0561400000 0.2139000000 0.4963000000	C C 0.0609000000 0.2011000000 0.4941000000
C C 0.0011300000 0.1448000000 0.4408000000	C C 0.0084000000 0.1290000000 0.4388000000
C C 0.0217000000 0.0498000000 0.4122000000	C C 0.0303000000 0.0374000000 0.4124000000
C C 0.0963000000 0.0230000000 0.4384000000	C C 0.1057000000 0.0130000000 0.4408000000
C C 0.1522500000 0.0914000000 0.4941000000	C C 0.1590000000 0.0837000000 0.4961000000
C C 0.2928500000 0.2361000000 0.6184800000	C C 0.2950000000 0.2330000000 0.6220000000
C C 0.3416900000 0.2297000000 0.7066000000	C C 0.3415000000 0.2273000000 0.7089000000
C C 0.4141800000 0.1909000000 0.7309000000	C C 0.4144000000 0.1900000000 0.7371000000
C C 0.4384400000 0.1583000000 0.6680000000	C C 0.4402000000 0.1587000000 0.6769000000
C C 0.3908400000 0.1658000000 0.5803000000	C C 0.3952000000 0.1660000000 0.5909000000
C C 0.3180700000 0.2046000000 0.5553600000	C C 0.3222000000 0.2028000000 0.5624000000
C C 0.2786100000 0.6647000000 0.6818900000	C C 0.2790000000 0.6603000000 0.6774000000
C C 0.3413900000 0.6719000000 0.7605000000	C C 0.3436000000 0.6687000000 0.7528000000
C C 0.3684900000 0.7721000000 0.7985000000	C C 0.3684000000 0.7697000000 0.7893000000
C C 0.3336000000 0.8675000000 0.7580000000	C C 0.3311000000 0.8639000000 0.7516000000

C C 0.2710000000 0.8610000000 0.6796000000	C C 0.2666000000 0.8551000000 0.6757000000
C C 0.2430100000 0.7607000000 0.6424000000	C C 0.2396000000 0.7544000000 0.6397000000
C C 0.3255000000 0.4834000000 0.5448200000	C C 0.3265000000 0.4781000000 0.5454000000
C C 0.3919700000 0.4806000000 0.6205000000	C C 0.3927000000 0.4773000000 0.6194000000
C C 0.4579800000 0.4443000000 0.6160000000	C C 0.4583000000 0.4439000000 0.6156000000
C C 0.4576100000 0.4108000000 0.5375000000	C C 0.4578000000 0.4107000000 0.5392000000
C C 0.3916500000 0.4131000000 0.4628000000	C C 0.3924000000 0.4100000000 0.4658000000
C C 0.3256800000 0.4487000000 0.4660400000	C C 0.3261000000 0.4445000000 0.4679000000
C C 0.1906500000 0.6164000000 0.4664800000	C C 0.1918000000 0.6110000000 0.4664000000
C C 0.2248500000 0.6670000000 0.4177800000	C C 0.2247000000 0.6633000000 0.4193000000
C C 0.1834300000 0.7377000000 0.3510000000	C C 0.1827000000 0.7343000000 0.3538000000
C C 0.1088000000 0.7583000000 0.3327000000	C C 0.1090000000 0.7533000000 0.3361000000
C C 0.0748800000 0.7098000000 0.3820900000	C C 0.0764000000 0.7038000000 0.3832000000
C C 0.1154400000 0.6391000000 0.4487900000	C C 0.1174000000 0.6319000000 0.4486000000
N N 0.1840700000 0.3163900000 0.6759600000	N N 0.1839000000 0.3121000000 0.6703000000
N N 0.2496100000 0.5591900000 0.6480400000	N N 0.2511000000 0.5534000000 0.6443000000
P P 0.1990400000 0.2839100000 0.5923500000	P P 0.2007800000 0.2783100000 0.5908900000
P P 0.2395200000 0.5224700000 0.5531100000	P P 0.2409500000 0.5163700000 0.5515900000
Mn Mn 0.1926600000 0.4808000000 0.7158300000	Mn Mn 0.1958700000 0.4761200000 0.7101800000
Br Br 0.0616100000 0.5504100000 0.6405800000	I I 0.0588200000 0.5584100000 0.6377700000
Br Br 0.2711300000 0.4829300000 0.8733300000	I I 0.2803200000 0.4771800000 0.8737500000
H H 0.4102391395 0.9183640624 0.6119962581	H H 0.4147645785 0.9036755469 0.6272436088
H H 0.4001344660 0.9179481818 0.4999203087	H H 0.3934361222 0.9192179556 0.5143828064
C C 0.4307000000 0.8845000000 0.5656000000	C C 0.4289000000 0.8791000000 0.5750000000
Cl Cl 0.4162600000 0.7432100000 0.5612000000	Cl Cl 0.4161600000 0.7381300000 0.5609700000
Cl Cl 0.5274500000 0.9173100000 0.6018100000	Cl Cl 0.5234600000 0.9162100000 0.6025800000
H H -0.1778194532 0.8758753409 0.0403630069	H H -0.1247055367 0.9219473994 -0.0075002406
H H -0.1225267319 0.9288311155 -0.0119963965	H H -0.1810838062 0.8672342322 0.0395044526
H H -0.2183979174 0.5996647786 -0.1911939038	H H -0.2172804326 0.5952121862 -0.1876825221
H H -0.1660792128 0.4618475858 -0.2569523480	H H -0.1675685764 0.4596237164 -0.2551749167
H H -0.0791610324 0.5173892765 -0.3250832938	H H -0.0860853462 0.5183220237 -0.3243535944
H H -0.0525164940 0.7128625399 -0.3335768382	H H -0.0591535101 0.7146592593 -0.3297197722
H H -0.1031830009 0.8521011844 -0.2654975665	H H -0.1058169662 0.8512274359 -0.2582646797
H H -0.0398354938 0.7893566426 -0.0184989785	H H -0.0423395396 0.7752875429 -0.0144989669
H H 0.0581235361 0.6665285922 0.0804311274	H H 0.0508489893 0.6480984327 0.0833288902
H H 0.0216562296 0.4955706797 0.1317452059	H H 0.0113693826 0.4816226922 0.1317435568
H H -0.1126922178 0.4484627878 0.0846829864	H H -0.1233563587 0.4391453250 0.0807722931
H H -0.2112770142 0.5697817983 -0.0133556469	H H -0.2181416129 0.5646511339 -0.0174613896
H H -0.3218123331 0.7568321293 -0.2553270560	H H -0.3201008334 0.7533655321 -0.2547696498
H H -0.4526072439 0.6872549834 -0.2996207934	H H -0.4511979047 0.6861378824 -0.3052173984
H H -0.4957454971 0.6277146623 -0.1875047179	H H -0.4974153958 0.6288171704 -0.1986542842
H H -0.4098106945 0.6413284265 -0.0302491842	H H -0.4161664089 0.6426801159 -0.0441413716
H H -0.2812003949 0.7085132088 0.0140010207	H H -0.2869762053 0.7069067784 0.0062835630
H H -0.3681240780 1.0966282660 -0.2932613005	H H -0.3734212118 1.0945800505 -0.2838895506
H H -0.4174785231 1.2743699320 -0.3607902000	H H -0.4186809506 1.2736474021 -0.3494885904
H H -0.3546276160 1.4467314854 -0.2873809317	H H -0.3511426775 1.4437710315 -0.2801204916
H H -0.2424155546 1.4348723517 -0.1460605355	H H -0.2360912084 1.4284213985 -0.1436447521
H H -0.1925856541 1.2570740475 -0.0817497914	H H -0.1876289406 1.2486515504 -0.0818546873
H H -0.3910679974 1.0056874441 -0.1821991858	H H -0.3923859592 1.0018963370 -0.1798991259
H H -0.5099242161 0.9412502240 -0.1751823231	H H -0.5103401826 0.9434964696 -0.1738179736
H H -0.5093260795 0.8820518051 -0.0339143036	H H -0.5095504824 0.8842490222 -0.0366289141
H H -0.3913262802 0.8855605602 0.0989447556	H H -0.3918878344 0.8823372312 0.0944403128
H H -0.2735928912 0.9492678841 0.0933278075	H H -0.2743296598 0.9444185338 0.0908501371
H H -0.2838572390 1.1514886021 0.0681016816	H H -0.2832103060 1.1487090089 0.0664932461
H H -0.2105859952 1.2772595422 0.1866311273	H H -0.2084778262 1.2749223203 0.1830552563
H H -0.0767774751 1.3133122347 0.2208549513	H H -0.0763669149 1.3084743306 0.2163700052
H H -0.0158138421 1.2262220380 0.1325463768	H H -0.0178593935 1.2194320380 0.1313314462
H H -0.0883764584 1.1018963209 0.0122355677	H H -0.0906510971 1.0931207682 0.0145052021
C C -0.1801800000 0.9029000000 -0.0232500000	C C -0.1824000000 0.8959000000 -0.0216000000
C C -0.1620700000 0.7351000000 -0.2204700000	C C -0.1623000000 0.7326000000 -0.2150000000
C C -0.1808600000 0.6253000000 -0.2212300000	C C -0.1811000000 0.6219000000 -0.2172000000
C C -0.1517200000 0.5475000000 -0.2588600000	C C -0.1537000000 0.5457000000 -0.2560000000
C C -0.1044600000 0.5782000000 -0.2981000000	C C -0.1091000000 0.5784000000 -0.2955000000
C C -0.0888100000 0.6877000000 -0.3016800000	C C -0.0933000000 0.6878000000 -0.2975000000

C C -0.1172400000 0.7661000000 -0.2639500000	C C -0.1196000000 0.7649000000 -0.2575000000
C C -0.1317600000 0.6871000000 -0.0228200000	C C -0.1366000000 0.6785000000 -0.0227000000
C C -0.0561400000 0.7139000000 0.0037000000	C C -0.0609000000 0.7011000000 0.0059000000
C C -0.0011300000 0.6448000000 0.0592000000	C C -0.0084000000 0.6290000000 0.0612000000
C C -0.0217000000 0.5498000000 0.0878000000	C C -0.0303000000 0.5374000000 0.0876000000
C C -0.0963000000 0.5230000000 0.0616000000	C C -0.1057000000 0.5130000000 0.0592000000
C C -0.1522500000 0.5914000000 0.0059000000	C C -0.1590000000 0.5837000000 0.0039000000
C C -0.2928500000 0.7361000000 -0.1184800000	C C -0.2950000000 0.7330000000 -0.1220000000
C C -0.3416900000 0.7297000000 -0.2066000000	C C -0.3415000000 0.7273000000 -0.2089000000
C C -0.4141800000 0.6909000000 -0.2309000000	C C -0.4144000000 0.6900000000 -0.2371000000
C C -0.4384400000 0.6583000000 -0.1680000000	C C -0.4402000000 0.6587000000 -0.1769000000
C C -0.3908400000 0.6658000000 -0.0803000000	C C -0.3952000000 0.6660000000 -0.0909000000
C C -0.3180700000 0.7046000000 -0.0553600000	C C -0.3222000000 0.7028000000 -0.0624000000
C C -0.2786100000 1.1647000000 -0.1818900000	C C -0.2790000000 1.1603000000 -0.1774000000
C C -0.3413900000 1.1719000000 -0.2605000000	C C -0.3436000000 1.1687000000 -0.2528000000
C C -0.3684900000 1.2721000000 -0.2985000000	C C -0.3684000000 1.2697000000 -0.2893000000
C C -0.3336000000 1.3675000000 -0.2580000000	C C -0.3311000000 1.3639000000 -0.2516000000
C C -0.2710000000 1.3610000000 -0.1796000000	C C -0.2666000000 1.3551000000 -0.1757000000
C C -0.2430100000 1.2607000000 -0.1424000000	C C -0.2396000000 1.2544000000 -0.1397000000
C C -0.3255000000 0.9834000000 -0.0448200000	C C -0.3265000000 0.9781000000 -0.0454000000
C C -0.3919700000 0.9806000000 -0.1205000000	C C -0.3927000000 0.9773000000 -0.1194000000
C C -0.4579800000 0.9443000000 -0.1160000000	C C -0.4583000000 0.9439000000 -0.1156000000
C C -0.4576100000 0.9108000000 -0.0375000000	C C -0.4578000000 0.9107000000 -0.0392000000
C C -0.3916500000 0.9131000000 0.0372000000	C C -0.3924000000 0.9100000000 0.0342000000
C C -0.3256800000 0.9487000000 0.0339600000	C C -0.3261000000 0.9445000000 0.0321000000
C C -0.1906500000 1.1164000000 0.0335200000	C C -0.1918000000 1.1110000000 0.0336000000
C C -0.2248500000 1.1670000000 0.0822200000	C C -0.2247000000 1.1633000000 0.0807000000
C C -0.1834300000 1.2377000000 0.1490000000	C C -0.1827000000 1.2343000000 0.1462000000
C C -0.1088000000 1.2583000000 0.1673000000	C C -0.1090000000 1.2533000000 0.1639000000
C C -0.0748800000 1.2098000000 0.1179100000	C C -0.0764000000 1.2038000000 0.1168000000
C C -0.1154400000 1.1391000000 0.0512100000	C C -0.1174000000 1.1319000000 0.0514000000
N N -0.1840700000 0.8163900000 -0.1759600000	N N -0.1839000000 0.8121000000 -0.1703000000
N N -0.2496100000 1.0591900000 -0.1480400000	N N -0.2511000000 1.0534000000 -0.1443000000
P P -0.1990400000 0.7839100000 -0.0923500000	P P -0.2007800000 0.7783100000 -0.0908900000
P P -0.2395200000 1.0224700000 -0.0531100000	P P -0.2409500000 1.0163700000 -0.0515900000
Mn Mn -0.1926600000 0.9808000000 -0.2158300000	Mn Mn -0.1958700000 0.9761200000 -0.2101800000
Br Br -0.0616100000 1.0504100000 -0.1405800000	I I -0.0588200000 1.0584100000 -0.1377700000
Br Br -0.2711300000 0.9829300000 -0.3733300000	I I -0.2803200000 0.9771800000 -0.3737500000
H H -0.4102391395 1.4183640624 -0.1119962581	H H -0.4147645785 1.4036755469 -0.1272436088
H H -0.4001344660 1.4179481818 0.0000796913	H H -0.3934361222 1.4192179556 -0.0143828064
C C -0.4307000000 1.3845000000 -0.0656000000	C C -0.4289000000 1.3791000000 -0.0750000000
Cl Cl -0.4162600000 1.2432100000 -0.0612000000	Cl Cl -0.4161600000 1.2381300000 -0.0609700000
Cl Cl -0.5274500000 1.4173100000 -0.1018100000	Cl Cl -0.5234600000 1.4162100000 -0.1025800000
H H -0.1778194532 -0.3758753409 -0.4596369931	H H -0.1247055367 -0.4219473994 -0.5075002406
H H -0.1225267319 -0.4288311155 -0.5119963965	H H -0.1810838062 -0.3672342322 -0.4604955474
H H -0.2183979174 -0.0996647786 -0.6911939038	H H -0.2172804326 -0.0952121862 -0.6876825221
H H -0.1660792128 0.0381524142 -0.7569523480	H H -0.1675685764 0.0403762836 -0.7551749167
H H -0.0791610324 -0.0173892765 -0.8250832938	H H -0.0860853462 -0.0183220237 -0.8243535944
H H -0.0525164940 -0.2128625399 -0.8335768382	H H -0.0591535101 -0.2146592593 -0.8297197722
H H -0.1031830009 -0.3521011844 -0.7654975665	H H -0.1058169662 -0.3512274359 -0.7582646797
H H -0.0398354938 -0.2893566426 -0.5184989785	H H -0.0423395396 -0.2752875429 -0.5144989669
H H 0.0581235361 -0.1665285922 -0.4195688726	H H 0.0508489893 -0.1480984327 -0.4166711098
H H 0.0216562296 0.0044293203 -0.3682547941	H H 0.0113693826 0.0183773078 -0.3682564432
H H -0.1126922178 0.0515372122 -0.4153170136	H H -0.1233563587 0.0608546750 -0.4192277069
H H -0.2112770142 -0.0697817983 -0.5133556469	H H -0.2181416129 -0.0646511339 -0.5174613896
H H -0.3218123331 -0.2568321293 -0.7553270560	H H -0.3201008334 -0.2533655321 -0.7547696498
H H -0.4526072439 -0.1872549834 -0.7996207934	H H -0.4511979047 -0.1861378824 -0.8052173984
H H -0.4957454971 -0.1277146623 -0.6875047179	H H -0.4974153958 -0.1288171704 -0.6986542842
H H -0.4098106945 -0.1413284265 -0.5302491842	H H -0.4161664089 -0.1426801159 -0.5441413716
H H -0.2812003949 -0.2085132088 -0.4859989793	H H -0.2869762053 -0.2069067784 -0.4937164370
H H -0.3681240780 -0.5966282660 -0.7932613005	H H -0.3734212118 -0.5945800505 -0.7838895506
H H -0.4174785231 -0.7743699320 -0.8607902000	H H -0.4186809506 -0.7736474021 -0.8494885904
H H -0.3546276160 -0.9467314854 -0.7873809317	H H -0.3511426775 -0.9437710315 -0.7801204916
H H -0.2424155546 -0.9348723517 -0.6460605355	H H -0.2360912084 -0.9284213985 -0.6436447521

H H -0.1925856541 -0.7570740475 -0.5817497914	H H -0.1876289406 -0.7486515504 -0.5818546873
H H -0.3910679974 -0.5056874441 -0.6821991858	H H -0.3923859592 -0.5018963370 -0.6798991259
H H -0.5099242161 -0.4412502240 -0.6751823231	H H -0.5103401826 -0.4434964696 -0.6738179736
H H -0.5093260795 -0.3820518051 -0.5339143036	H H -0.5095504824 -0.3842490222 -0.5366289141
H H -0.3913262802 -0.3855605602 -0.4010552444	H H -0.3918878344 -0.3823372312 -0.4055596872
H H -0.2735928912 -0.4492678841 -0.4066721925	H H -0.2743296598 -0.4444185338 -0.4091498629
H H -0.2838572390 -0.6514886021 -0.4318983184	H H -0.2832103060 -0.6487090089 -0.4335067539
H H -0.2105859952 -0.7772595422 -0.3133688727	H H -0.2084778262 -0.7749223203 -0.3169447437
H H -0.0767774751 -0.8133122347 -0.2791450487	H H -0.0763669149 -0.8084743306 -0.2836299948
H H -0.0158138421 -0.7262220380 -0.3674536232	H H -0.0178593935 -0.7194320380 -0.3686685538
H H -0.0883764584 -0.6018963209 -0.4877644323	H H -0.0906510971 -0.5931207682 -0.4854947979
C C -0.1801800000 -0.4029000000 -0.5232500000	C C -0.1824000000 -0.3959000000 -0.5216000000
C C -0.1620700000 -0.2351000000 -0.7204700000	C C -0.1623000000 -0.2326000000 -0.7150000000
C C -0.1808600000 -0.1253000000 -0.7212300000	C C -0.1811000000 -0.1219000000 -0.7172000000
C C -0.1517200000 -0.0475000000 -0.7588600000	C C -0.1537000000 -0.0457000000 -0.7560000000
C C -0.1044600000 -0.0782000000 -0.7981000000	C C -0.1091000000 -0.0784000000 -0.7955000000
C C -0.0888100000 -0.1877000000 -0.8016800000	C C -0.0933000000 -0.1878000000 -0.7975000000
C C -0.1172400000 -0.2661000000 -0.7639500000	C C -0.1196000000 -0.2649000000 -0.7575000000
C C -0.1317600000 -0.1871000000 -0.5228200000	C C -0.1366000000 -0.1785000000 -0.5227000000
C C -0.0561400000 -0.2139000000 -0.4963000000	C C -0.0609000000 -0.2011000000 -0.4941000000
C C -0.0011300000 -0.1448000000 -0.4408000000	C C -0.0084000000 -0.1290000000 -0.4388000000
C C -0.0217000000 -0.0498000000 -0.4122000000	C C -0.0303000000 -0.0374000000 -0.4124000000
C C -0.0963000000 -0.0230000000 -0.4384000000	C C -0.1057000000 -0.0130000000 -0.4408000000
C C -0.1522500000 -0.0914000000 -0.4941000000	C C -0.1590000000 -0.0837000000 -0.4961000000
C C -0.2928500000 -0.2361000000 -0.6184800000	C C -0.2950000000 -0.2330000000 -0.6220000000
C C -0.3416900000 -0.2297000000 -0.7066000000	C C -0.3415000000 -0.2273000000 -0.7089000000
C C -0.4141800000 -0.1909000000 -0.7309000000	C C -0.4144000000 -0.1900000000 -0.7371000000
C C -0.4384400000 -0.1583000000 -0.6680000000	C C -0.4402000000 -0.1587000000 -0.6769000000
C C -0.3908400000 -0.1658000000 -0.5803000000	C C -0.3952000000 -0.1660000000 -0.5909000000
C C -0.3180700000 -0.2046000000 -0.5553600000	C C -0.3222000000 -0.2028000000 -0.5624000000
C C -0.2786100000 -0.6647000000 -0.6818900000	C C -0.2790000000 -0.6603000000 -0.6774000000
C C -0.3413900000 -0.6719000000 -0.7605000000	C C -0.3436000000 -0.6687000000 -0.7528000000
C C -0.3684900000 -0.7721000000 -0.7985000000	C C -0.3684000000 -0.7697000000 -0.7893000000
C C -0.3336000000 -0.8675000000 -0.7580000000	C C -0.3311000000 -0.8639000000 -0.7516000000
C C -0.2710000000 -0.8610000000 -0.6796000000	C C -0.2666000000 -0.8551000000 -0.6757000000
C C -0.2430100000 -0.7607000000 -0.6424000000	C C -0.2396000000 -0.7544000000 -0.6397000000
C C -0.3255000000 -0.4834000000 -0.5448200000	C C -0.3265000000 -0.4781000000 -0.5454000000
C C -0.3919700000 -0.4806000000 -0.6205000000	C C -0.3927000000 -0.4773000000 -0.6194000000
C C -0.4579800000 -0.4443000000 -0.6160000000	C C -0.4583000000 -0.4439000000 -0.6156000000
C C -0.4576100000 -0.4108000000 -0.5375000000	C C -0.4578000000 -0.4107000000 -0.5392000000
C C -0.3916500000 -0.4131000000 -0.4628000000	C C -0.3924000000 -0.4100000000 -0.4658000000
C C -0.3256800000 -0.4487000000 -0.4660400000	C C -0.3261000000 -0.4445000000 -0.4679000000
C C -0.1906500000 -0.6164000000 -0.4664800000	C C -0.1918000000 -0.6110000000 -0.4664000000
C C -0.2248500000 -0.6670000000 -0.4177800000	C C -0.2247000000 -0.6633000000 -0.4193000000
C C -0.1834300000 -0.7377000000 -0.3510000000	C C -0.1827000000 -0.7343000000 -0.3538000000
C C -0.1088000000 -0.7583000000 -0.3327000000	C C -0.1090000000 -0.7533000000 -0.3361000000
C C -0.0748800000 -0.7098000000 -0.3820900000	C C -0.0764000000 -0.7038000000 -0.3832000000
C C -0.1154400000 -0.6391000000 -0.4487900000	C C -0.1174000000 -0.6319000000 -0.4486000000
N N -0.1840700000 -0.3163900000 -0.6759600000	N N -0.1839000000 -0.3121000000 -0.6703000000
N N -0.2496100000 -0.5591900000 -0.6480400000	N N -0.2511000000 -0.5534000000 -0.6443000000
P P -0.1990400000 -0.2839100000 -0.5923500000	P P -0.2007800000 -0.2783100000 -0.5908900000
P P -0.2395200000 -0.5224700000 -0.5531100000	P P -0.2409500000 -0.5163700000 -0.5515900000
Mn Mn -0.1926600000 -0.4808000000 -0.7158300000	Mn Mn -0.1958700000 -0.4761200000 -0.7101800000
Br Br -0.0616100000 -0.5504100000 -0.6405800000	I I -0.0588200000 -0.5584100000 -0.6377700000
Br Br -0.2711300000 -0.4829300000 -0.8733300000	I I -0.2803200000 -0.4771800000 -0.8737500000
H H -0.4102391395 -0.9183640624 -0.6119962581	H H -0.4147645785 -0.9036755469 -0.6272436088
H H -0.4001344660 -0.9179481818 -0.4999203087	H H -0.3934361222 -0.9192179556 -0.5143828064
C C -0.4307000000 -0.8845000000 -0.5656000000	C C -0.4289000000 -0.8791000000 -0.5750000000
Cl Cl -0.4162600000 -0.7432100000 -0.5612000000	Cl Cl -0.4161600000 -0.7381300000 -0.5609700000
Cl Cl -0.5274500000 -0.9173100000 -0.6018100000	Cl Cl -0.5234600000 -0.9162100000 -0.6025800000
H H 0.1778194532 0.1241246591 0.9596369931	H H 0.1247055367 0.0780526006 1.0075002406
H H 0.1225267319 0.0711688845 1.0119963965	H H 0.1810838062 0.1327657678 0.9604955474
H H 0.2183979174 0.4003352214 1.1911939038	H H 0.2172804326 0.4047878138 1.1876825221
H H 0.1660792128 0.5381524142 1.2569523480	H H 0.1675685764 0.5403762836 1.2551749167

H H 0.0791610324 0.4826107235 1.3250832938	H H 0.0860853462 0.4816779763 1.3243535944
H H 0.0525164940 0.2871374601 1.3335768382	H H 0.0591535101 0.2853407407 1.3297197722
H H 0.1031830009 0.1478988156 1.2654975665	H H 0.1058169662 0.1487725641 1.2582646797
H H 0.0398354938 0.2106433574 1.0184989785	H H 0.0423395396 0.2247124571 1.0144989669
H H -0.0581235361 0.3334714078 0.9195688726	H H -0.0508489893 0.3519015673 0.9166711098
H H -0.0216562296 0.5044293203 0.8682547941	H H -0.0113693826 0.5183773078 0.8682564432
H H 0.1126922178 0.5515372122 0.9153170136	H H 0.1233563587 0.5608546750 0.9192277069
H H 0.2112770142 0.4302182017 1.0133556469	H H 0.2181416129 0.4353488661 1.0174613896
H H 0.3218123331 0.2431678707 1.2553270560	H H 0.3201008334 0.2466344679 1.2547696498
H H 0.4526072439 0.3127450166 1.2996207934	H H 0.4511979047 0.3138621176 1.3052173984
H H 0.4957454971 0.3722853377 1.1875047179	H H 0.4974153958 0.3711828296 1.1986542842
H H 0.4098106945 0.3586715735 1.0302491842	H H 0.4161664089 0.3573198841 1.0441413716
H H 0.2812003949 0.2914867912 0.9859989793	H H 0.2869762053 0.2930932216 0.9937164370
H H 0.3681240780 -0.0966282660 1.2932613005	H H 0.3734212118 -0.0945800505 1.2838895506
H H 0.4174785231 -0.2743699320 1.3607902000	H H 0.4186809506 -0.2736474021 1.3494885904
H H 0.3546276160 -0.4467314854 1.2873809317	H H 0.3511426775 -0.4437710315 1.2801204916
H H 0.2424155546 -0.4348723517 1.1460605355	H H 0.2360912084 -0.4284213985 1.1436447521
H H 0.1925856541 -0.2570740475 1.0817497914	H H 0.1876289406 -0.2486515504 1.0818546873
H H 0.3910679974 -0.0056874441 1.1821991858	H H 0.3923859592 -0.0018963370 1.1798991259
H H 0.5099242161 0.0587497760 1.1751823231	H H 0.5103401826 0.0565035304 1.1738179736
H H 0.5093260795 0.1179481949 1.0339143036	H H 0.5095504824 0.1157509778 1.0366289141
H H 0.3913262802 0.1144394398 0.9010552444	H H 0.3918878344 0.1176627688 0.9055596872
H H 0.2735928912 0.0507321159 0.9066721925	H H 0.2743296598 0.0555814662 0.9091498629
H H 0.2838572390 -0.1514886021 0.9318983184	H H 0.2832103060 -0.1487090089 0.9335067539
H H 0.2105859952 -0.2772595422 0.8133688727	H H 0.2084778262 -0.2749223203 0.8169447437
H H 0.0767774751 -0.3133122347 0.7791450487	H H 0.0763669149 -0.3084743306 0.7836299948
H H 0.0158138421 -0.2262220380 0.8674536232	H H 0.0178593935 -0.2194320380 0.8686685538
H H 0.0883764584 -0.1018963209 0.9877644323	H H 0.0906510971 -0.0931207682 0.9854947979
C C 0.1801800000 0.0971000000 1.0232500000	C C 0.1824000000 0.1041000000 1.0216000000
C C 0.1620700000 0.2649000000 1.2204700000	C C 0.1623000000 0.2674000000 1.2150000000
C C 0.1808600000 0.3747000000 1.2212300000	C C 0.1811000000 0.3781000000 1.2172000000
C C 0.1517200000 0.4525000000 1.2588600000	C C 0.1537000000 0.4543000000 1.2560000000
C C 0.1044600000 0.4218000000 1.2981000000	C C 0.1091000000 0.4216000000 1.2955000000
C C 0.0888100000 0.3123000000 1.3016800000	C C 0.0933000000 0.3122000000 1.2975000000
C C 0.1172400000 0.2339000000 1.2639500000	C C 0.1196000000 0.2351000000 1.2575000000
C C 0.1317600000 0.3129000000 1.0228200000	C C 0.1366000000 0.3215000000 1.0227000000
C C 0.0561400000 0.2861000000 0.9963000000	C C 0.0609000000 0.2989000000 0.9941000000
C C 0.0011300000 0.3552000000 0.9408000000	C C 0.0084000000 0.3710000000 0.9388000000
C C 0.0217000000 0.4502000000 0.9122000000	C C 0.0303000000 0.4626000000 0.9124000000
C C 0.0963000000 0.4770000000 0.9384000000	C C 0.1057000000 0.4870000000 0.9408000000
C C 0.1522500000 0.4086000000 0.9941000000	C C 0.1590000000 0.4163000000 0.9961000000
C C 0.2928500000 0.2639000000 1.1184800000	C C 0.2950000000 0.2670000000 1.1220000000
C C 0.3416900000 0.2703000000 1.2066000000	C C 0.3415000000 0.2727000000 1.2089000000
C C 0.4141800000 0.3091000000 1.2309000000	C C 0.4144000000 0.3100000000 1.2371000000
C C 0.4384400000 0.3417000000 1.1680000000	C C 0.4402000000 0.3413000000 1.1769000000
C C 0.3908400000 0.3342000000 1.0803000000	C C 0.3952000000 0.3340000000 1.0909000000
C C 0.3180700000 0.2954000000 1.0553600000	C C 0.3222000000 0.2972000000 1.0624000000
C C 0.2786100000 -0.1647000000 1.1818900000	C C 0.2790000000 -0.1603000000 1.1774000000
C C 0.3413900000 -0.1719000000 1.2605000000	C C 0.3436000000 -0.1687000000 1.2528000000
C C 0.3684900000 -0.2721000000 1.2985000000	C C 0.3684000000 -0.2697000000 1.2893000000
C C 0.3336000000 -0.3675000000 1.2580000000	C C 0.3311000000 -0.3639000000 1.2516000000
C C 0.2710000000 -0.3610000000 1.1796000000	C C 0.2666000000 -0.3551000000 1.1757000000
C C 0.2430100000 -0.2607000000 1.1424000000	C C 0.2396000000 -0.2544000000 1.1397000000
C C 0.3255000000 0.0166000000 1.0448200000	C C 0.3265000000 0.0219000000 1.0454000000
C C 0.3919700000 0.0194000000 1.1205000000	C C 0.3927000000 0.0227000000 1.1194000000
C C 0.4579800000 0.0557000000 1.1160000000	C C 0.4583000000 0.0561000000 1.1156000000
C C 0.4576100000 0.0892000000 1.0375000000	C C 0.4578000000 0.0893000000 1.0392000000
C C 0.3916500000 0.0869000000 0.9628000000	C C 0.3924000000 0.0900000000 0.9658000000
C C 0.3256800000 0.0513000000 0.9660400000	C C 0.3261000000 0.0555000000 0.9679000000
C C 0.1906500000 -0.1164000000 0.9664800000	C C 0.1918000000 -0.1110000000 0.9664000000
C C 0.2248500000 -0.1670000000 0.9177800000	C C 0.2247000000 -0.1633000000 0.9193000000
C C 0.1834300000 -0.2377000000 0.8510000000	C C 0.1827000000 -0.2343000000 0.8538000000
C C 0.1088000000 -0.2583000000 0.8327000000	C C 0.1090000000 -0.2533000000 0.8361000000
C C 0.0748800000 -0.2098000000 0.8820900000	C C 0.0764000000 -0.2038000000 0.8832000000

C C 0.1154400000 -0.1391000000 0.9487900000	C C 0.1174000000 -0.1319000000 0.9486000000
N N 0.1840700000 0.1836100000 1.1759600000	N N 0.1839000000 0.1879000000 1.1703000000
N N 0.2496100000 -0.0591900000 1.1480400000	N N 0.2511000000 -0.0534000000 1.1443000000
P P 0.1990400000 0.2160900000 1.0923500000	P P 0.2007800000 0.2216900000 1.0908900000
P P 0.2395200000 -0.0224700000 1.0531100000	P P 0.2409500000 -0.0163700000 1.0515900000
Mn Mn 0.1926600000 0.0192000000 1.2158300000	Mn Mn 0.1958700000 0.0238800000 1.2101800000
Br Br 0.0616100000 -0.0504100000 1.1405800000	I I 0.0588200000 -0.0584100000 1.1377700000
Br Br 0.2711300000 0.0170700000 1.3733300000	I I 0.2803200000 0.0228200000 1.3737500000
H H 0.4102391395 -0.4183640624 1.1119962581	H H 0.4147645785 -0.4036755469 1.1272436088
H H 0.4001344660 -0.4179481818 0.9999203087	H H 0.3934361222 -0.4192179556 1.0143828064
C C 0.4307000000 -0.3845000000 1.0656000000	C C 0.4289000000 -0.3791000000 1.0750000000
Cl Cl 0.4162600000 -0.2432100000 1.0612000000	Cl Cl 0.4161600000 -0.2381300000 1.0609700000
Cl Cl 0.5274500000 -0.4173100000 1.1018100000	Cl Cl 0.5234600000 -0.4162100000 1.1025800000
#END	#END

[a] The positions of the hydrogen atoms in the crystals were optimized by means of periodic DFT calculations, carried out with the PBEsol DFT functional [5] in combination with on-the-fly generated pseudopotentials [6]. The planewave basis set cut-off was set at 1000 eV. The dispersion corrections from Tkatchenko and Scheffler were added [7]. Relativistic effects were accounted through the scalar Koelling–Harmon approximation [8]. The positions of the other atoms and the periodic boundary conditions were taken from the X-ray data and kept fixed. The cell contents considered in the calculations were $H_{136}C_{152}N_8P_8Cl_8Mn_4X_8$ (X = Br, I), corresponding to 1076 (X = Br) and 996 (X = I) explicit electrons. The charge was set to zero and the number of unpaired electrons in the unit cell was set to twenty, according to the presence of four high-spin manganese(II) centres. The software used was CASTEP version 23.1 [9,10].

[5] Perdew, J.P.; Ruzsinszky, A.; Csonka, G.I.; Vydrov, O.A.; Scuseria, G.E.; Constantin, L.A.; Zhou, X.; Burke, K. Restoring the Density-Gradient Expansion for Exchange in Solids and Surfaces. *Phys. Rev. Lett.* **2008**, *100*, 136406. [6] Lin, J.S.; Qteish, A.; Payne, M.C.; Heine, V. Optimized and transferable nonlocal separable ab initio pseudopotentials. *Phys. Rev. B* **1993**, *47*, 4174–4180. [7] Tkatchenko, A.; Scheffler, M. Accurate Molecular Van Der Waals Interactions from Ground-State Electron Density and Free-Atom Reference Data. *Phys. Rev. Lett.* **2009**, *102*, 073005. [8] Koelling, D.D.; Harmon, B.N. A technique for relativistic spin-polarised calculations. *J. Phys. C Solid State Phys.* **1977**, *10*, 3107–3114. [9] Clark, S.J.; Segall, M.D.; Pickard, C.J.; Hasnip, P.J.; Probert, M.I.J.; Refson, K.; Payne, M.C. First principles methods using CASTEP. *Z. Krist.* **2005**, *220*, 567–570. [10] Rutter, M.J. C2x: A tool for visualisation and input preparation for Castep and other electronic structure codes. *Comput. Phys. Commun.* **2018**, *225*, 174–179.

Table S5. Cartesian coordinates of the DFT-optimized geometries of [MnX_2{(PhN=PPh_2)CH_2}].

**X = Br, sextet**				**X = I, sextet**			
76				76			
MnC37P2N2H32Br2-sextet				MnC37P2N2H32I2-sextet			
Mn	-1.560381000	5.952693000	10.748209000	Mn	-1.607437000	5.869063000	10.944341000
Br	-3.433763000	6.724676000	9.281940000	I	-3.614079000	6.756911000	9.343183000
Br	-1.240945000	5.733004000	13.163060000	I	-1.157488000	5.728585000	13.551261000
P	-0.391594000	3.535709000	8.998270000	P	-0.495391000	3.424597000	9.183186000
P	0.675771000	6.360173000	8.363664000	P	0.598574000	6.243727000	8.537209000
N	-1.442686000	3.917921000	10.109270000	N	-1.513391000	3.840990000	10.316349000
N	0.292118000	6.670522000	9.869519000	N	0.191613000	6.589015000	10.027296000
C	-0.327894000	4.949925000	7.855121000	C	-0.387148000	4.821649000	8.026908000
H	0.023725000	4.613894000	6.881492000	H	-1.417250000	5.161268000	7.887511000
H	-1.359966000	5.298219000	7.757723000	H	-0.000686000	4.461644000	7.074867000
C	-2.136975000	2.955249000	10.865699000	C	-2.234251000	2.884192000	11.059406000
C	-1.563934000	1.755429000	11.276263000	C	-1.704207000	1.648477000	11.418307000
H	-0.533710000	1.539477000	11.030272000	H	-0.689492000	1.396480000	11.144150000
C	-2.295141000	0.850631000	12.027982000	C	-2.459112000	0.747593000	12.150758000
H	-1.833395000	-0.074755000	12.342269000	H	-2.030514000	-0.206648000	12.422563000
C	-3.600563000	1.134471000	12.390818000	C	-3.745610000	1.070380000	12.547138000
H	-4.165826000	0.433255000	12.986778000	H	-4.329418000	0.370944000	13.126943000

C	-4.171112000	2.333870000	11.989043000	C	-4.272736000	2.305245000	12.198354000
H	-5.187184000	2.571040000	12.269745000	H	-5.273808000	2.573064000	12.503860000
C	-3.451757000	3.234762000	11.228179000	C	-3.529753000	3.202785000	11.456109000
H	-3.896678000	4.163806000	10.900713000	H	-3.947155000	4.156748000	11.166084000
C	-0.932340000	2.142684000	8.008884000	C	-1.118276000	2.058224000	8.204280000
C	-2.128801000	2.311853000	7.313583000	C	-2.380661000	2.243089000	7.641409000
H	-2.649299000	3.258736000	7.354708000	H	-2.910034000	3.174370000	7.792133000
C	-2.671248000	1.256807000	6.605140000	C	-2.971354000	1.220707000	6.923431000
H	-3.600363000	1.389532000	6.071217000	H	-3.951024000	1.364130000	6.492750000
C	-2.030626000	0.026137000	6.597395000	C	-2.312095000	0.009460000	6.771814000
H	-2.459603000	-0.800196000	6.049544000	H	-2.777613000	-0.790830000	6.215215000
C	-0.853090000	-0.151388000	7.304972000	C	-1.066868000	-0.183271000	7.347248000
H	-0.366126000	-1.115315000	7.315371000	H	-0.563307000	-1.133316000	7.245930000
C	-0.303218000	0.904247000	8.013002000	C	-0.468901000	0.838512000	8.065928000
H	0.601502000	0.757625000	8.582624000	H	0.489834000	0.675927000	8.533747000
C	1.263207000	3.220523000	9.606227000	C	1.157530000	3.047438000	9.756607000
C	1.493236000	3.518427000	10.945475000	C	1.407863000	3.263933000	11.107445000
H	0.689534000	3.909792000	11.555828000	H	0.623335000	3.647650000	11.746720000
C	2.751863000	3.311827000	11.485827000	C	2.665330000	2.992908000	11.622105000
H	2.929374000	3.540856000	12.526085000	H	2.857449000	3.157694000	12.671832000
C	3.770940000	2.807628000	10.695277000	C	3.663168000	2.508293000	10.793400000
H	4.750322000	2.640989000	11.120282000	H	4.641093000	2.290616000	11.198116000
C	3.545092000	2.520840000	9.355674000	C	3.419303000	2.309663000	9.440833000
H	4.345918000	2.140740000	8.739338000	H	4.205491000	1.949643000	8.794411000
C	2.294371000	2.730530000	8.808334000	C	2.170034000	2.582901000	8.919692000
H	2.122587000	2.515645000	7.763653000	H	1.986301000	2.437212000	7.865076000
C	0.906730000	7.746811000	10.568734000	C	0.631374000	7.795374000	10.645862000
C	1.545411000	7.481446000	11.776437000	C	1.454337000	7.730105000	11.764586000
H	1.536615000	6.472649000	12.158060000	H	1.733873000	6.762626000	12.153003000
C	2.150994000	8.502818000	12.485131000	C	1.858298000	8.889049000	12.401918000
H	2.634703000	8.281097000	13.425541000	H	2.487742000	8.822178000	13.277412000
C	2.128132000	9.803111000	12.003903000	C	1.440947000	10.126514000	11.937259000
H	2.601034000	10.599255000	12.559851000	H	1.752914000	11.030134000	12.439902000
C	1.471684000	10.074472000	10.815189000	C	0.595184000	10.192980000	10.842186000
H	1.423727000	11.086890000	10.440182000	H	0.237353000	11.149709000	10.489997000
C	0.855590000	9.057516000	10.104171000	C	0.183251000	9.035828000	10.202651000
H	0.318727000	9.283817000	9.196643000	H	-0.508312000	9.086178000	9.376069000
C	2.393765000	5.910026000	8.085038000	C	2.325352000	5.797543000	8.310598000
C	3.318047000	6.034548000	9.112509000	C	3.189909000	5.827218000	9.394177000
H	2.999069000	6.382569000	10.081296000	H	2.812474000	6.098046000	10.365906000
C	4.647223000	5.715708000	8.886526000	C	4.527809000	5.513819000	9.218340000
H	5.363373000	5.819138000	9.688041000	H	5.199837000	5.545072000	10.062889000
C	5.053591000	5.262379000	7.643653000	C	4.998472000	5.157016000	7.966552000
H	6.090811000	5.013993000	7.471028000	H	6.041934000	4.911618000	7.831575000
C	4.130279000	5.125695000	6.616198000	C	4.133609000	5.115694000	6.881202000
H	4.445318000	4.771664000	5.645508000	H	4.501697000	4.840259000	5.903776000
C	2.806124000	5.455019000	6.833946000	C	2.802197000	5.444436000	7.050204000
H	2.096582000	5.371767000	6.021980000	H	2.137387000	5.442950000	6.197193000
C	0.350144000	7.646228000	7.146496000	C	0.325981000	7.520128000	7.301952000
C	1.383299000	8.340653000	6.522941000	C	1.368810000	8.380217000	6.964839000
H	2.411070000	8.073792000	6.713620000	H	2.356062000	8.220092000	7.370680000
C	1.094447000	9.391117000	5.668937000	C	1.138844000	9.450638000	6.119746000
H	1.899452000	9.929652000	5.190885000	H	1.949062000	10.118560000	5.867552000
C	-0.222623000	9.754057000	5.435811000	C	-0.130118000	9.666944000	5.604967000
H	-0.445377000	10.575661000	4.770416000	H	-0.308644000	10.503985000	4.945635000
C	-1.253912000	9.072447000	6.064978000	C	-1.172403000	8.817629000	5.942723000
H	-2.280955000	9.362651000	5.900187000	H	-2.165682000	8.992664000	5.557218000
C	-0.972748000	8.024545000	6.922380000	C	-0.950106000	7.749518000	6.793163000
H	-1.781699000	7.531773000	7.447050000	H	-1.782871000	7.130735000	7.093293000
**X = Br, octet**				**X = I, octet**			
76				76			
MnC37P2N2H32Br2-octet				MnC37P2N2H32I2-octet			
Mn	-1.375353000	6.018022000	11.007871000	Mn	-1.544411000	5.949920000	11.133918000
Br	-3.422695000	6.745883000	9.819994000	I	-3.815545000	6.621612000	9.863083000

Br	-1.043646000	5.748800000	13.420317000	I	-1.122778000	5.873626000	13.754776000
P	-0.324457000	3.442992000	9.005009000	P	-0.468879000	3.355150000	9.159288000
P	0.542273000	6.350903000	8.439875000	P	0.402456000	6.257552000	8.580197000
N	-1.416560000	3.872141000	10.306446000	N	-1.560600000	3.801748000	10.464698000
N	0.324658000	6.725881000	9.970117000	N	0.142265000	6.652861000	10.098018000
C	-0.581680000	4.999647000	8.068379000	C	-0.708056000	4.899313000	8.199230000
H	-0.458669000	4.764199000	7.014008000	H	-1.730159000	5.271345000	8.329904000
H	-1.597092000	5.377534000	8.228250000	H	-0.557961000	4.647522000	7.152243000
C	-2.225641000	2.985459000	10.885734000	C	-2.360938000	2.911064000	11.053394000
C	-2.154796000	1.576055000	10.663188000	C	-2.311519000	1.505035000	10.804801000
H	-1.420936000	1.175442000	9.983717000	H	-1.600134000	1.106989000	10.100674000
C	-3.004442000	0.727493000	11.320615000	C	-3.157707000	0.653732000	11.463271000
H	-2.929155000	-0.336494000	11.152237000	H	-3.099755000	-0.407143000	11.270232000
C	-3.963875000	1.225487000	12.206537000	C	-4.091921000	1.144377000	12.379469000
H	-4.628756000	0.544909000	12.717943000	H	-4.756083000	0.461983000	12.889378000
C	-4.065696000	2.598605000	12.429072000	C	-4.168400000	2.513190000	12.634119000
H	-4.811267000	2.983908000	13.107950000	H	-4.892345000	2.894824000	13.337982000
C	-3.218919000	3.465615000	11.791410000	C	-3.325385000	3.382202000	11.994195000
H	-3.306977000	4.527893000	11.951849000	H	-3.396611000	4.441059000	12.183686000
C	-0.516484000	2.178001000	7.852023000	C	-0.648286000	2.083579000	8.007274000
C	-1.475251000	2.314953000	6.801139000	C	-1.594200000	2.216352000	6.945170000
H	-1.975509000	3.258813000	6.640690000	H	-2.086659000	3.161595000	6.769818000
C	-1.794403000	1.250466000	5.993872000	C	-1.911656000	1.146269000	6.144214000
H	-2.513479000	1.394920000	5.199509000	H	-2.620467000	1.288028000	5.340240000
C	-1.216608000	-0.005850000	6.186563000	C	-1.344260000	-0.111479000	6.355254000
H	-1.476820000	-0.836190000	5.548842000	H	-1.602554000	-0.946076000	5.722280000
C	-0.317169000	-0.172928000	7.245949000	C	-0.457964000	-0.274290000	7.425518000
H	0.124987000	-1.143193000	7.425755000	H	-0.023405000	-1.245394000	7.618683000
C	0.015429000	0.869243000	8.072072000	C	-0.127616000	0.774018000	8.245521000
H	0.696898000	0.701363000	8.892987000	H	0.543964000	0.609827000	9.075258000
C	1.226144000	3.268511000	9.858633000	C	1.076833000	3.182605000	10.022532000
C	1.382497000	3.702310000	11.175711000	C	1.225958000	3.622954000	11.338019000
H	0.551274000	4.108908000	11.732426000	H	0.393752000	4.039806000	11.884950000
C	2.618058000	3.607379000	11.792040000	C	2.454600000	3.522681000	11.966842000
H	2.725181000	3.947689000	12.811570000	H	2.553091000	3.868096000	12.985644000
C	3.699468000	3.070223000	11.114692000	C	3.537455000	2.972550000	11.302440000
H	4.660798000	2.995079000	11.601991000	H	4.493461000	2.891023000	11.799111000
C	3.546691000	2.629174000	9.805549000	C	3.393128000	2.526919000	9.993848000
H	4.387792000	2.215187000	9.269541000	H	4.235956000	2.104288000	9.467401000
C	2.326141000	2.740946000	9.175140000	C	2.179462000	2.644670000	9.351205000
H	2.213517000	2.425927000	8.149837000	H	2.074619000	2.325641000	8.326294000
C	1.043763000	7.780167000	10.595020000	C	0.791901000	7.765184000	10.705257000
C	1.694953000	7.519759000	11.797319000	C	1.579629000	7.548750000	11.831147000
H	1.629104000	6.530896000	12.221522000	H	1.682099000	6.543109000	12.207333000
C	2.395777000	8.521214000	12.444720000	C	2.196642000	8.610229000	12.467459000
H	2.887918000	8.302436000	13.381414000	H	2.795536000	8.426655000	13.347714000
C	2.463435000	9.796052000	11.903893000	C	2.038010000	9.901768000	11.989313000
H	3.011899000	10.576353000	12.410582000	H	2.517977000	10.730678000	12.488303000
C	1.802627000	10.062797000	10.716216000	C	1.241445000	10.122301000	10.877794000
H	1.827952000	11.056663000	10.292565000	H	1.092677000	11.126679000	10.508047000
C	1.090112000	9.067924000	10.067448000	C	0.612592000	9.064513000	10.241659000
H	0.555848000	9.296414000	9.159244000	H	-0.032769000	9.247205000	9.397270000
C	2.198285000	5.805796000	8.024344000	C	2.069166000	5.712810000	8.207101000
C	3.252891000	6.063985000	8.890764000	C	3.098946000	5.955790000	9.105977000
H	3.072553000	6.568673000	9.826034000	H	2.891627000	6.446822000	10.042573000
C	4.534778000	5.665675000	8.550643000	C	4.390340000	5.563898000	8.795032000
H	5.352775000	5.869552000	9.225290000	H	5.189306000	5.756564000	9.495296000
C	4.763919000	5.000044000	7.358586000	C	4.652348000	4.918939000	7.598565000
H	5.764567000	4.686070000	7.098937000	H	5.659745000	4.608903000	7.361309000
C	3.710666000	4.730503000	6.496445000	C	3.623298000	4.665992000	6.702397000
H	3.886211000	4.204584000	5.569606000	H	3.825080000	4.157742000	5.771058000
C	2.431294000	5.138210000	6.824181000	C	2.335580000	5.069093000	7.000951000
H	1.617597000	4.934504000	6.142814000	H	1.541454000	4.881924000	6.292182000
C	0.145650000	7.632630000	7.242056000	C	0.055000000	7.536836000	7.367277000

C	1.105316000	8.176097000	6.394335000	C	1.079690000	8.171106000	6.672275000
H	2.118268000	7.804413000	6.411563000	H	2.102378000	7.853352000	6.805259000
C	0.762541000	9.207146000	5.535515000	C	0.788138000	9.220897000	5.817684000
H	1.510283000	9.630647000	4.881225000	H	1.586373000	9.714965000	5.283713000
C	-0.533476000	9.696862000	5.521425000	C	-0.522962000	9.637839000	5.653426000
H	-0.797251000	10.502806000	4.851924000	H	-0.747825000	10.457742000	4.986723000
C	-1.491176000	9.159735000	6.369908000	C	-1.547023000	9.009098000	6.347436000
H	-2.499911000	9.545419000	6.367655000	H	-2.568851000	9.337587000	6.228345000
C	-1.155753000	8.133151000	7.232842000	C	-1.262126000	7.964776000	7.207081000
H	-1.898919000	7.738969000	7.914648000	H	-2.061734000	7.505749000	7.773904000
